# Selective Functionalization of Carbonyl *Closo*-Decaborate [2-B_10_H_9_CO]^−^ with Building Block Properties via Grignard Reagents

**DOI:** 10.3390/molecules28166076

**Published:** 2023-08-15

**Authors:** Nadine Mahfouz, Fatima Abi-Ghaida, Wael Kotob, Ahmad Mehdi, Daoud Naoufal

**Affiliations:** 1 Inorganic and Organometallic Coordination Chemistry Laboratory LCIO, Faculty of Sciences, Lebanese University, Beirut P.O. Box 6573, Lebanon; nadine.mahfouz@etu.umontpellier.fr (N.M.); wael.kotob@ul.edu.lb (W.K.); 2 Institut Charles Gerhardt ICGM, CNRS, ENSCM, Université de Montpellier, 34090 Montpellier, France

**Keywords:** hydroborate, *closo*-decaborate, Grignard reagents RMgX, building blocks [2-B_10_H_9_COR]^2−^

## Abstract

A green, fast and selective approach for the synthesis of mono-substituted *closo*-decaborate derivatives [2-B_10_H_9_COR]^2−^ has been established via a nucleophilic addition reaction between the carbonyl derivative of *closo*-decaborate [2-B_10_H_9_CO]^−^ and the corresponding Grignard reagent RMgX, where R is the ethyl, iso-propyl, pentyl, allyl, vinyl and propynyl groups. This approach is accomplished under mild conditions with 70–80% yields. The significance of these derivative is their ability to constitute building blocks for polymeric integration via the allyl, vinyl and propynyl substituents. All products were characterized by ^11^B, ^1^H and ^13^C NMR, elemental analysis and mass spectrometry.

## 1. Introduction

The *closo*-borate anions [B_n_H_n_]^2−^ (n = 6–12), particularly the *closo*-decaborate [B_10_H_10_]^2−^, represent one of the most multifaceted and appealing compounds in polyhedral architecture [1,2,3]. Their physiochemical properties, such as thermodynamic stability [4], electrochemical properties [5,6], three-dimensional aromaticity [3], modulated biological compatibility [7,8], as well as their tunable hydrophobicity have encouraged their integration in a wide spectrum of applications ranging from metal complexation [9], medicine [7] and catalysis [10] to material sciences [11,12,13], solid-state batteries [14,15,16] and hydrogen storage [17,18]. Functionalized or derivatized compounds of the polyhedral boron clusters are often regarded as versatile and circumventive building blocks, particularly in the biomedical field. Indeed, the pharmacological aptitudes of these three-dimensional inorganic cages are often comparable to that of the three-dimensional diamondoid cages of adamantine but with far more desired electronic properties that infer their derivatives with stabilizing properties [19]. For example, reactive nitrilium derivatives of the *closo*-borate anions often retain their stability and structural integrity under mild aerobic, acidic or basic conditions, which render them suitable for in situ physiological applications [20]. Pharmacologically oriented research on boron clusters currently focuses on their coalescence within self-sufficient systems of diagnostics and treatment; their unique features as selective receptor agonists are preferable to classical organic scaffolds and are frequently exploited in further elucidation of structure–activity relationship (SAR) profiles [21]. One of the long-standing medicinal applications of boron clusters is boron neutron capture therapy (BNCT), a preferential and theoretically infallible targeted therapy regime that exploits boron-10 isotopes’ susceptibility to thermal neutrons [22]. The BNCT protocol or, more precisely, the boron-10-enriched functionalized clusters suffer from nonpreferential and inadequate accumulation within the tumor cells due to the lack of selective carriers; hence, a universally and medically acquiesced BNCT protocol demands establishing a synergistic unified system comprising boron-10 sources and targeting carriers. Recently, Yorov et al. immobilized an alkoxysilane derivative bearing a 10-vertex *closo*-decaborate nanocluster in a silica (SiO_2_) aerogel matrix. The silica nanomaterial loaded with 1.2 mol % *closo*-decaborate possessed high specific surface area (740 m^2^/g), low apparent density (80 mg/cm^3^) and exhibited low toxicity towards normal cells and considerable cytotoxicity towards malignant glioblastoma cells [23]. The strategy was first introduced in 2014, where the authors reported the first-in-class borate-alkoxysilane derivatives via the functionalization of the carbonyl- and diazo-derivatives of *closo*-decaborate and validated the integrity/activity of the precursors to integrate into the matrix, pores and surfaces of silica-based nanomaterial (biologically compatible mesoporous silica and silica nanoparticles) as proof of concept [24,25]. Another synergistic carrier was introduced by Stepanova et al. to combine boron delivery to the tumor cells of osteosarcoma and the repair of postoperative bone defects; the authors reported boron-containing scaffolds comprising novel biodegradable polymer composites as films and 3D-printed matrices based on aliphatic polyesters containing *closo*-borates clusters for BNCT [26].

**Figure 1:**
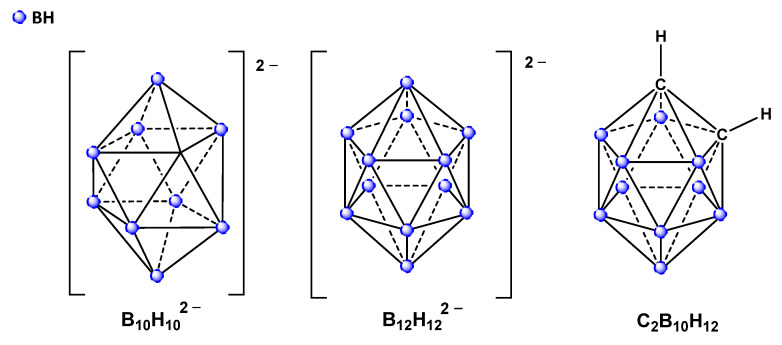
Schematic representation of the *closo*-decaborate anion [B_10_H_10_]^2−^ (**left**), *closo*-dodecaborate anion [B_12_H_12_]^2−^ (**middle**) and the neutral icosahedral carborane C_2_B_10_H_12_ (**right**).

The impediment in integrating inorganic boron-based polyhedral archetypes such as the anionic *closo*-decaborate [B_10_H_10_]^2−^ and the *closo*-dodecaborate [B_12_H_12_]^2−^ into industrial or pharmacological venues lies in the introduction of functional groups into theses clusters [27,28,29,30,31]. Indeed, the ability to engineer functional boron-enriched materials via derivatization of the clusters, or what is rather known as the activation of one or more exo-polyhedral B-H bonds, is often constrained for compounds with a purely boron skeleton as opposed to their neutral carbon-enclosing analogs as the icosahedral dicarba-*closo*-dodecaborane [C_2_B_10_H_12_] [32]. The polyhedral carboranes rather possess an intrinsic advantage that fundamentally bridges the chemistry of boron clusters with the vast and established chemistry of carbon-based scaffolds, the B-C-H bonds (Figure 1); thus, these compounds have become quite favored in research and industry and have been profoundly investigated in establishing antitumor, antimicrobial and antiviral formulations [33] via their integration into the structure of existing conventional drugs or prodrugs to alter SAR profiles [34,35]. The functionalization pathways of carboranes are quite vast and established; the most recent approach reported direct B–H functionalization of icosahedral carboranes at the most electron-rich boron vertex, that is, the boron vertex with the lowest B–H bond dissociation energy, where a nitrogen-centered radical-mediated hydrogen atom transfer instigated the homolysis of the B–H bond [36]. Conventionally recognized pathways include Metal-catalyzed cross-coupling reactions for assembling larger molecules via covalently bonded molecular fragments [37], the Sonogashira [38], Heck [39] and Suzuki cross-coupling reactions [40], the Kumada-Corriu cross-coupling reactions between B-iodo-carboranes and Grignard reagents in the presence of palladium-based catalysts [41]. A nucleophilic substitution Grignard reaction pathway was also established for B–H bond activation of ortho-carboranes in the absence of any transition metal catalysts; however, the reaction necessitates the presence of two electron-withdrawing aryl groups on the cage carbon atoms [42].

The impedance in the functionalization of the *closo*-decaborate anion is primarily dictated by the electronic environment of the cage; this can be quite challenging due to the presence of 10 inert B–H bonds in a rather stable and comparable chemical environment; B-H bond substitution can proceed via electrophilic or nucleophilic mechanisms in either apical (boron atoms with a co-ordination number of 4) or equatorial (boron atoms with a co-ordination number of 5) positions to yield mono-, di- and poly-substituted derivatives [27,30,31,43]. A recent soft approach utilized an auto-catalyzed reaction pathway to functionalize (NH_4_)_2_[B_10_H_10_] by exploiting the in situ NH_4_^+^ counter cation during the nucleophilic addition of nitriles to the borate cluster via Electrophilic-Induced Nucleophilic Substitution mechanism [44,45].

**Figure 2:**
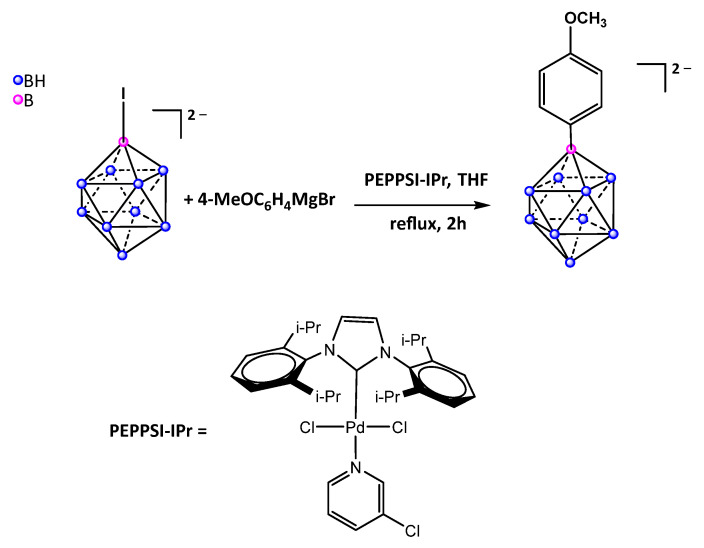
Synthesis of *closo*-decaborate derivative encompassing a direct B-C(Ph) bond via the iodo-*closo*-decaborate precursor.

One of the foremost derivatives of the *closo*-decaborate anion is the carbonyl protagonist [1-B_10_H_9_CO]^−^ [46], which has been repeatedly employed as a precursor for further functionalization of the decaborate cage. The carbonyl-mediated reaction pathway has been presented as an alternative to direct alkylation of the *closo*-decaborate, which predominantly necessitates manipulations with nido-decaborane B_10_H_14_ under unfavorable conditions and yields poly-substituted derivatives. Recently, a direct alkylation strategy of the B–H bond in *closo*-decaborate was reported by Kaszynski et al. following a Pd-catalyzed cross-coupling reaction approach via iodo precursors which has been extensively applied for substitutions of 12-vertex carboranes and dodecaborate anion [47]. Establishing a direct B–C bond in [B_10_H_10_]^2−^ has mainly been hindered by the lack of access to suitable precursors such as the iodo-derivatives of decaborate anion, which exhibits a practically diminutive reactivity toward iodination (Figure 2). The authors attempted to synthesize the [*closo*-1,10-B_10_H_8_-I_2_]^2–^ anion from the bis-iodonium zwitterion [*closo*-1,10-B_10_H_8_-(IPh)_2_] through Grignard reagents (proven successful for carboranes as the [*closo*-CB_11_H_11_I]^−^) but isolation proved ineffective due to the formation of insoluble magnesium salts and instead diverted to organolithium reagents.

Though the carbonyl derivative is prone to facile and rapid hydrolysis leading to the carboxylic derivative [1-B_10_H_9_COOH]^2−^ and its manipulation normally requires extremely anhydrous and inert conditions, it is still the preferable precursor of choice due to the versatility supplied by the carbonyl group. Moreover, new reaction pathways for the selective functionalization of this derivative are currently under investigation, as conventional approaches suffer from nonselective side reactions as it is easily susceptible to a wide range of functional groups [48,49]. Thus, the main notion of the present work is to establish a selective synthesis route starting from the carbonyl derivative of *closo*-decaborate to compounds decorated with alkyl chains, such as ethyl, propyl and pentyl chains, as well as building block “polymerizable” or “conjugative” constituents, such as the allyl, vinyl and propynyl derivatives. The necessity of such alkylated and conjugable boron clusters is frequently noted for biological applications such as antimicrobial applications [50] and boron neutron capture therapy; indeed, fabricating such derivatives enclosing a hydrophilic charged head (anionic boron cluster) and a hydrophobic tail (alkyl chain) render them as ideal candidates for incorporation into physiological media such as lipophilic bilayers and uni-lamellar liposome penetration, particularly as liposomes have been proven to preferentially localize in tumor cells. Currently, boron-loaded liposomes are under investigation as self-sufficient systems for BNCT cancer treatment [51].

## 2. Results and Discussion

Herein, we report the facile and selective synthesis of versatile *closo*-decaborate derivatives comprising alkyl chains as the propyl and pentyl chains and building blocks as the allyl, vinyl and propynyl groups. The novel synthesis approach proceeds via a straightforward nucleophilic addition reaction at room temperature between the carbonyl derivative of *closo*-decaborate (PPh_4_) [B_10_H_9_CO] and a Grignard reagent RMgX comprising the desired functionality under an inert atmosphere.

**Figure 3:**
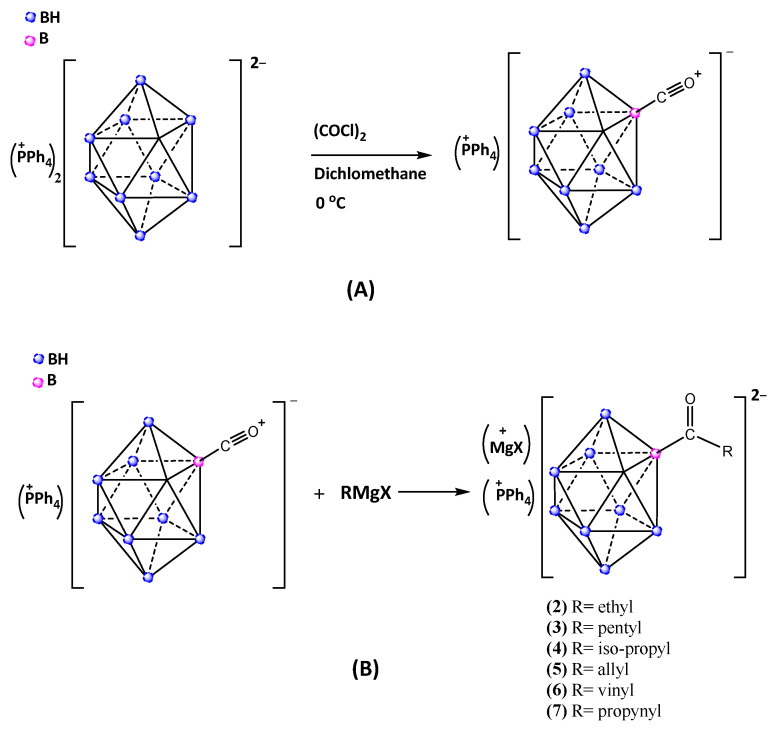
Schematic representation of the synthesis of carbonyl-*closo*-decaborate (**A**) and the nucleophilic addition of Grignard reagents RMgX to the carbonyl derivative of *closo*-decaborate (**B**).

First, the tetraphenyl phosphonium salt of the precursor compound carbonyl-*closo*-decaborate (PPh_4_)[2-B_10_H_9_CO] was prepared according to published procedures by the reaction of (PPh_4_)_2_[B_10_H_10_] with drop-wise addition of excess of oxalyl chloride (COCl)_2_ in anhydrous dichloromethane at 0 °C as seen in Figure 3A [46].

Second, six different Grignard reagents were used, ranging from simple alkyl reagents, such as ethyl, iso-propyl and pentyl, to the more versatile allyl, vinyl and propynyl groups as depicted in Figure 3B; the last three of these groups encompass building block functionalities for further conjugation or polymerization of the *closo*-decaborate cluster into functional materials.

The straightforward nucleophilic addition of the alkyl Grignard reagents comprising ethyl, iso-propyl and pentyl magnesium bromide to the carbonyl derivative yields the corresponding carbonyl-alkyl or acyl derivatives [2-B_10_H_9_C(O)CH_2_CH_3_]^2−^ (**2**), [2-B_10_H_9_C(O)C_3_H_7_]^2−^ (**3**) and [2-B_10_H_9_C(O)C_5_H_9_]^2−^ (**4**), isolated by mere precipitation out of the reaction mixture via diethyl ether and filtration.

**Figure 4:**
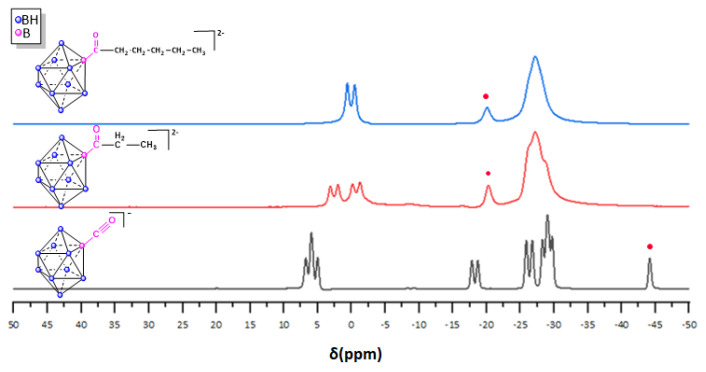
^11^B NMR spectrum of carbonyl-*closo*-decaborate derivative (PPh_4_) [2-B_10_H_9_C≡O] (**1**), (PPh_4_)(MgBr) [2-B_10_H_9_C(O)CH_2_CH_3_] (**2**), (PPh_4_)(MgBr) [2-B_10_H_9_C(O)C_5_H_9_] (**3**).

The structural integrity of the *closo*-derivatives was verified through multinuclear ^1^H, ^11^B, ^13^C and ^31^P NMR. The ^11^B NMR spectra of all derivatives exhibit a downfield shift in the singlet characteristic of the carbonyl precursor (PPh_4_)[2-B_10_H_9_C≡O] at −44.71 ppm to approximately −20 ppm for the carbonyl-alkyl derivatives; for instance, the ^11^B NMR of the two alkyl-substituted derivatives (PPh_4_)(MgBr)[2-B_10_H_9_C(O)CH_2_CH_3_] (**2**) and (PPh_4_)(MgBr)[2-B_10_H_9_C(O)C_5_H_9_] (**3**) confirm the appearance of a singlet at −20.21 for (**2**) and at −19.98 for (**3**) and the disappearance of the equatorially carbonyl-substituted boron B−C≡O^+^ at −44.7 ppm consistent with the appearance of the electron-withdrawing C=O bonds in place of the C≡O^+^ group, which, in turn, contributes to a further deshielding of the boron atom (Figure 4). Furthermore, the ^11^B NMR of the two derivatives [2-B_10_H_9_C(O)CH_2_CH_3_]^2−^ (**2**) and [2-B_10_H_9_C(O)C_5_H_9_]^2−^ (**3**) evidences the apical boron atoms as two doublets at 2.59 ppm (B_1_) and −0.77 (B_10_) ppm for (**2**) and one doublet at 0.23 ppm (B_1,10_), while the remaining equatorial boron atoms appear as overlapping broad multiplets at ca. −28 ppm (see Figure S1 in the ESI for ^11^B NMR of all compounds).

^1^H and ^13^C NMR of the derivatives (PPh_4_)(MgBr)[2-B_10_H_9_C(O)CH_2_CH_3_] (**2**), (PPh_4_)(MgBr)[2-B_10_H_9_C(O)C_5_H_9_] (**3**) and (PPh_4_)(MgBr)[2-B_10_H_9_C(O)C_3_H_7_] (**4**) display the characteristic peaks of the carbonyl, ethyl, isopropyl and pentyl groups. ^1^H NMR (see Figure S2 in ESI) of [2-B_10_H_9_C(O)CH_2_CH_3_]^2−^ exhibits a triplet at 0.65 ppm for CH_3_, a quadruplet at 2.23 ppm for CH_2_ and a multiplet between 7.69 and 7.99 for the tetraphenyl phosphonium hydrogens (PPh_4_); further evidence on the tetraphenyl phosphonium counter cation is seen in ^31^P NMR as a singlet at 22.59 ppm, while the ^13^C NMR spectrum exhibits a peak at 157.30 ppm for the carbonyl group C=O, a peak for the sp^3^ carbon in −CH_2_**C**H_3_ at 1.13 ppm and another peak at 7.25 ppm for **C**H_2_CH_3_; the aromatic carbons in tetraphenyl phosphonium appear similarly for all derivatives at 118.49 (doublet resulting from carbon–phosphorous coupling), 130.45 (ortho-C), 134.88 (meta-C) and 135.52 (para-C). Similarly, the ^1^H NMR of the isopropyl-substituted derivative [2-B_10_H_9_C(O)C_3_H_7_]^2−^ (**4**) displays a doublet at 0.68 ppm for the two CH_3_ groups (see Figure S10 in the ESI), a sextuplet at 2.65 ppm for CH and a multiplet between 7.60 and 8.19 for the tetraphenyl phosphonium cation. The ^13^C NMR (see Figure S11 in the ESI) shows a peak at 19.59 ppm for the sp^3^ carbon in CH_3_. As for pentyl-substituted derivative [2-B_10_H_9_C_5_H_9_]^2−^ (**3**), the ^1^H NMR (see Figure S6 in the ESI) spectrum exhibits a triplet at 0.75 ppm for CH_3_, a sextuplet at 1.10 ppm for CH_2_, a quintuplet at 1.25 ppm for CH_2_ and a triplet at 2.15 ppm for C(O)CH_2;_ a multiplet spanning the range of 7.69 and 8.20 evidences the tetraphenyl phosphonium counter cation. The counter cation PPh_4_^+^ was also detected by the appearance of a singlet in the ^31^P NMR at 22.13 ppm affirming, the presence of phosphonium ion in compound (**2**) (see Figure S4 in the ESI). In addition, the ^13^C NMR (see Figure S7 in the ESI) exhibits a peak at 166.35 ppm for the carbonyl group, a peak at 14.50 ppm for the sp^3^ carbon in -CH_3_ and other peaks at 22.73 ppm, 24.01 ppm, 32.08 ppm and 45.29 ppm for the -CH_2_ in the compound.

As previously mentioned, direct alkylation or acylation of the *closo*-decaborate cage has not yet been reported; though the chemistry of the *closo*-decaborate cage is quite versatile, its derivatization or functionalization approaches are often restricted by the higher stability and rigidity of the exopolyhedral B–H bonds in [B_10_H_10_]^2–^ compared to other polyhedral boranes. Introduction of a functional group into the decaborate moiety conventionally occurs by means of functionalized precursors (interim compounds) such as the carbonyl precursor [2-B_10_H_9_C≡O]^−^, diazonium precursor [1-B_10_H_9_N≡N]^−^ and nitrilium precursor [2-B_10_H_9_N≡CCH_3_]^−^. Hence, [2-B_10_H_9_C≡O]^−^ was chosen as an ideal candidate for the expansion of the decaborate chemistry. To assert the necessity of the carbonyl group C≡O^+^ in the present work (i.e., prove that direct alkylation of the decaborate cage via Grignard reagents is not possible under present conditions) and to further elucidate the nucleophilic addition mechanism, several control reactions were performed between the parent compound (the tetraphenylphosphonium salt of the *closo*-decaborate anion (PPh_4_)_2_[B_10_H_10_]), the protonated compound (undecahydro-*closo*-decaborate anion (PPh_4_)[B_10_H_11_]) and the corresponding Grignard reagents following an identical synthetic protocol to that stated in the experimental details. ^11^B NMR indicated the absence of any functionalization or degradation of the parent decaborate cluster for the reaction of the *closo*-decaborate anion [B_10_H_10_]^2−^ with RMgBr; in fact, the obtained data suggest the mere precipitation of the cage. Moreover, the second series of control reaction performed via the protonated undecahydro-*closo*-decaborate anion [B_10_H_11_]^−^ and RMgBr reagents suggest the deprotonation of the *closo*-decaborate cage and a complexation reaction taking place between [B_10_H_10_]^2−^ and the divalent metallic center Mg^2+^; the ^11^B NMR spectrum of the resulting isolated solid displays a downfield shift in the boron atom peaks residing in the apical position to ca. 3 ppm and another downfield peak shift of equatorial boron atoms to ca. −26 ppm (the ^11^B NMR peaks of (PPh_4_) [B_10_H_10_] is typically present at ca. 0 ppm for the two apical boron atoms and ca. −30 ppm for the eight equatorial boron atoms) while noting the disappearance of the protonated peak for [B_10_H_11_]^−^ (note that the chemical shifts observed for the resulting solid are compatible with magnesium-boron cage derivatives currently under investigation in hydrogen storage). The results can be further interpreted by examining the mechanism of action where Grignard reagents often react with protonated sources or acidic protons to yield the R−H compounds.

An identical synthesis methodology was used to functionalize the *closo*-decaborate carbonyl derivative [2-B_10_H_9_C≡O]^−^ with potential building block substituents comprising the allyl, vinyl and propynyl functionalities. The fairly straightforward synthesis involved the preparation of an etherate solution of (PPh_4_)[B_10_H_9_CO] in anhydrous THF under argon, followed by the corresponding dropwise addition of the Grignard reagent (allyl magnesium bromide, vinyl magnesium bromide and propynyl magnesium bromide). Isolation of the respective products via simple filtration and vacuum drying yielded the phosphonium and MgBr salts of [2-B_10_H_9_C(O)CH_2_CH=CH_2_]^2−^ (**5**), [2-B_10_H_9_C(O)CH=CH_2_]^2−^(**6**) and [2-B_10_H_9_C(O)C≡CCH_3_]^2−^ (**7**) derivatives, respectively. ^11^B NMR data of all derivatives clearly display a shift in the carbonyl singlet of −C≡O^+^ from the characteristic −44.71 ppm to −20.17, −18.62 and −18.22 ppm for the allyl −COCH_2_CH=CH_2_, vinyl −COCH=CH_2_ and propynyl −COC≡CCH_3_ derivatives (see Figure S1 in the ESI). The slight discrepancy in chemical shifts can be explained by the deshielding effect exerted by the vinyl and propynyl groups in their respective derivatives and can be explicitly attributed to the electronic resonance between the double and triple bonds of the vinyl −COCH=CH_2_ and propynyl −COC≡CCH_3_ groups and the oxygen lone pairs. ^1^H NMR spectra clearly display the characteristic peaks of the corresponding allyl and vinyl groups where the -CH=CH_2_ in [2- B_10_H_9_C(O)CH_2_CH=CH_2_]^2−^ (**5**) appears at 4.85 and 5.85 ppm. As for the vinyl group in the compound [2-B_10_H_9_C(O)CH=CH_2_]^2−^ (**6**), the ^1^H NMR spectrum displays the appearance of characteristic peaks at 4.95 ppm for −COCH=C**H_2_** and 6.49 ppm for −COC**H**=CH_2_, while the ^13^C NMR spectrum displays two peaks at 130.91 ppm (−COCH=**C**H_2_) and 135.95 ppm (−CO**C**H=CH_2_) and a small peak at 185.56 for the carbonyl group. For the [2-B_10_H_9_C(O)C≡CCH_3_]^2−^ (**7**) derivative, the structural information was confirmed by ^1^H NMR and ^13^C NMR; ^1^H NMR spectrum displays the appearance of a singlet at 1.75 ppm for the −COC≡CC**H_3_**, while ^13^C NMR shows the appearance of three peaks at 25.66 ppm for −COC≡C**C**H_3_, 67.50 ppm for −CO**C**≡CCH_3_ and 82.95 ppm for −COC≡**C**CH_3_.

## 3. Materials and Methods

All reactions were performed under an inert atmosphere (Argon) using vacuum tube and Schlenk techniques. All solvents used in the syntheses were dried and distilled accordingly. (Et_3_NH)_2_[B_10_H_10_] was purchased from Boron Specialties (Ambridge, PA, USA) and the salt (PPh_4_)_2_B_10_H_10_ was precipitated from an aqueous solution of (Et_3_NH)_2_B_10_H_10_ and recrystallized from an acetonitrile–Et_2_O mixture. Ethyl magnesium bromide, pentyl magnesium bromide, iso-propyl magnesium chloride, allyl magnesium chloride, vinyl magnesium bromide and 1-propynyl magnesium bromide were purchased from Sigma-Aldrich and used as received. Oxalyl chloride was obtained as 2.0 M solution in CH_2_Cl_2_ from Aldrich. Solution ^1^H NMR, ^13^C NMR and ^31^P NMR spectra were recorded using an AMX 400 Bruker spectrometer operating, respectively, at 400 MHz, 100 MHz and 162 MHz. For the analysis of our boron-based products, the ^11^B sequence used has the following characteristics: ^11^B spectra were recorded at 298 K on a Bruker Advance III 500 Mz NMR spectrometer equipped with a BBO helium cryoprobe. The ^11^B zgbs pulse sequence was used with a spectral width of 64,102 Hz and 256 scans with a relaxation time of 0.5 s. Chemical shifts were externally calibrated to TMS for ^1^H and 13C nuclei, H_3_PO_4_ (85%) for ^31^P nuclei and EtO_2_·BF_3_ for ^11^B nuclei. Deuterated DMSO and acetonitrile were used as solvents. Mass spectrometry measurements were performed by negative electrospray ionization method (ESI/MS).

### 3.1. Synthesis of Carbonyl-*Closo*-Decaborate (PPh_4_) [B_10_H_9_CO] (***1***)

(PPh_4_) [2-B_10_H_9_CO] (1) was prepared according to the published literature; in brief, a solution of (PPh_4_)_2_[B_10_H_10_] (1.346 g, 2 mmol) in 30 mL of dry dichloromethane was placed under argon at 0 °C. An excess of a 2.0 M solution of (COCl)_2_ in dichloromethane (2.5 mmol) was added dropwise and the mixture stirred for 30 min and then allowed to warm up to room temperature and stirred for an additional hour. The volume of the resulting mixture was reduced till ca. 3 mL, exposed to air and passed through a silica column and eluted with dry dichloromethane to reduce the hydrolysis of the −C≡O^+^. The resulting product was isolated with 74% yield after crystallization from CH_2_Cl_2_–Et_2_O. **^11^B (^1^H) NMR** (δ ppm, 128 MHz, CD_3_CN): 5.37 (d, 2B), −18.75 (d, 1B), −26.84 (d, 2B), −28.13 (d, 2B), −29.88 (d, 2B), −44.71 (s, 1B).

### 3.2. Synthesis of *Closo*-Decaborate Derivatives (PPh_4_)(MgX)[B_10_H_9_COR]

A solution containing 200 mg of (PPh_4_) [2-B_10_H_9_CO] and 10 mL of anhydrous THF was placed under argon at room temperature; one equivalent of the corresponding Grignard reagent RMgX was added dropwise under stirring. The reaction progress was monitored periodically by TLC (DEAE-Cellulose), where the complete disappearance of the carbonyl derivative was noted after 10 min of stirring for all derivatives. The reaction volume was reduced by 2/3 under vacuum, approximately 5 mL of anhydrous diethyl ether was added under dynamic argon flow and the mixture was placed at −20 °C for 1 to 2 h; the targeted products precipitated out of the mixture and were isolated as pale-yellow solids via simple filtration. The final solids were subjected to three cycles of washing with 5 mL of cold diethyl ether and dried under vacuum to yield (PPh_4_)(MgX)[B_10_H_9_COR], where R is the ethyl, pentyl, iso-propyl, allyl, vinyl and propynyl groups.

*(PPh_4_)(MgBr)[2-B_10_H_9_C(O)CH_2_CH_3_]* (**2**) **^11^B (^1^H) NMR** (δ ppm, 128 MHz, CD_3_CN): 2.59 (d, 1 B), −0.77 (d, 1 B), −20.21 (s, 1 B), between −25.39 and −29.41 (m, broad, 7 B). **^1^H NMR** (δ ppm, DMSO-*d*_6_): 0.65 (3 H, t, *J* = 8 Hz, CH_3_), 2.23 (2 H, quadruplet, *J* = 6 Hz, CH_2_), 7.5–7.8 (20 H, m, H of PPh_4_^+^). **^13^C NMR** (δ ppm, DMSO-*d*_6_): 157.30, 134.16, 133.32, 129.31, 116.49, (^1^J_C-P_ = 77 Hz), 7.25, 1.13. **^31^P NMR** (DMSO-*d*_6_): 22.58. **Mass spectrometry (ESI/MS):** *m*/*z* = 172.19 **Elemental analysis:** % theoretical C: 52.51. Found C: 52.26. **Yield:** 85%.

*(PPh_4_)(MgBr)[2-B_10_H_9_C(O)C_5_H_9_]* (**3**) **^11^B (^1^H) NMR** (δ ppm, 128 MHz, CD_3_CN): 0.23 (d, 2 B), −19.98 (s, 1B), between −24.75 and −29.40 (m, broad, 7B). **^1^H NMR** (δ ppm, DMSO-*d*_6_): 0.75 (3H, t, *J* = 6 Hz, CH_3_), 1.10 (2 H, sextuplet, *J* = 4 Hz, CH_2_), 1.25 (4 H, quintuplet, *J* = 4 Hz, 2CH_2_), 2.15 (2 H, t, *J* = 6 Hz, CH_2_), 7.5–7.8 (20 H, m, H of PPh_4_^+^).**^13^C NMR** (DMSO-*d*_6_): 166.35, 135.85, 135.01, 131.01, 118.14 (^1^J_C-P_ = 71 Hz), 32.08, 24.01, 22.73, 14.50. **^31^P NMR** (DMSO-*d*_6_): 22.13. **Mass spectrometry (ESI/MS):** *m*/*z* = 216. **Elemental analysis:** % theoretical C: 54.63. Found C: 54.25. **Yield:** 80%.

*(PPh_4_)(MgCl)[2-B_10_H_9_C(O)C_3_H_7_]* (**4**) **^11^B (^1^H) NMR** (δ ppm, 128 MHz, CD_3_CN): 3.30 (d, 2 B), −19.10 (s, 1 B), between −21.94 and −28.42 (m, broad, 7 B). **^1^H NMR** (δ ppm, DMSO-*d*_6_): 0.68 (6 H, d, *J* = 8 Hz, 2CH_3_), 2.65 (1 H, septuplet, *J* = 4 Hz, CH), 7.51–8.04 (20 H, m, H of PPh_4_^+^). ^13^C NMR (δ ppm, DMSO-*d*_6_): 135.86, 135.00, 131.01, 118.13 (^1^J_C-P_ = 71 Hz), 19.79. **^31^P NMR** (DMSO-*d*_6_): 22.56. **Mass spectrometry (ESI/MS):** *m*/*z* = 188. **Elemental analysis:** % theoretical C: 57.25. Found C: 57.35. **Yield:** 75%.

*(PPh_4_)(MgCl) [2-B_10_H_9_C(O)CH_2_CH=CH_2_]* (**5**) **^11^B (^1^H) NMR** (δ ppm, 128 MHz, CD_3_CN): 2.56 (d, 1 B), −0.51 (d, 1B) −20.17 (s, 1B), between −26 and −28 (m, broad, 7B). **^1^H NMR** (δ ppm, DMSO-*d*_6_): 3.25 (2H, d, *J* = 6 Hz, CH_2_), 4.85 (2H, m, CH_2_), 5.85 (1H, m, CH), 7.5–7.8 (20H, m, H of PPh_4_^+^). **^13^C NMR** (δ ppm, DMSO-*d*_6_):164.24, 135.86, 135.08, 131.01, 130.84, 118.13 (^1^J_C-P_ = 71 Hz), 45.95. **^31^P NMR** (DMSO-*d*_6_): 21.64. **Mass spectrometry (ESI/MS):** *m*/*z* = 185.20. **Elemental analysis:** % theoretical C: 57.45. Found C: 57.35. **Yield:** 68%.

*(PPh_4_)(MgBr) [2-B_10_H_9_C(O)CH=CH_2_]* (**6**) **^11^B (^1^H) NMR** (δ ppm, 128 MHz, CD_3_CN): 3.78 (d, 1B), 0.83 (d, 1B), −18.62 (s, 1B), between −25.54 and −31.32 (m, broad, 7 B). **^1^H NMR** (δ ppm, DMSO-*d*_6_): 4.95 (2H, m, CH_2_), 6.49 (1 H, m, CH), 7.5–7.8 (20 H, m H of PPh_4_^+^).**^13^C NMR** (δ ppm, DMSO-*d*_6_): 185.56, 135.85, 135.09, 134.90, 130.91, 118.14 (^1^J_C-P_ = 71 Hz). **^31^P NMR** (DMSO-*d*_6_): 22.14. **Mass spectrometry (ESI/MS):** *m*/*z* = 171.18. **Elemental analysis:** % theoretical C:52.68. Found C: 52.45. **Yield:** 73%.

*(PPh_4_)(MgBr) [2-B_10_H_9_C(O)C≡CCH_3_]* (**7**) **^11^B (^1^H) NMR** (δ ppm, 128 MHz, CD_3_CN): 3.05 (d, 1 B), −0.64 (d, 1B), −18.22 (s, 1B), between −25.28 and −29.06 (m, broad, 7 B). **^1^H NMR** (δ ppm, DMSO-*d*_6_): 1.75 (3 H, s, CH_3_), 7.5–7.8 (20 H, m, H of PPh_4_^+^). **^13^C NMR** (δ ppm, DMSO-*d*_6_): 171.20, 135.87, 135.00, 131.02, 118.13 (^1^J_C-P_ = 71 Hz), 82.95, 67.50, 25.66. **^31^P NMR** (DMSO-*d*_6_): 22.13. **Mass spectrometry (ESI/MS):** *m*/*z* = 184. **Elemental analysis:** % theoretical C: 53.57. Found C: 52.67. **Yield:** 70%.

## 4. Conclusions

In the present work, a selective synthetic approach to actively functionalize the *closo*-decaborate cage [B_10_H_10_]^2−^ with building block properties was developed. The approach centers on the exploitation of the carbonyl derivative [2-B_10_H_9_C≡O]^−^ as a starting precursor and subjecting that precursor to a straightforward nucleophilic addition reaction with a series of Grignard reagents of different R constituents. The approach was validated to the Grignard reagents used in this work but can be further extended to include all conceivable “RMgX” reagents, where R can be engineered as the desirable moiety; the developed strategy can be applied to embed or integrate the *closo*-decaborate cage in complex material systems such as porphyrins, metal organic frameworks, dendrimers, etc. The key derivatives achieved in the current work [2-B_10_H_9_C(O)CH=CH_2_]^2−^, [2-B_10_H_9_C(O)CH_2_CH=CH_2_]^2−^ and [2-B_10_H_9_C(O)C≡CCH_3_]^2−^ comprise the allyl, vinyl and propynyl functionalities and allow the conjugation/polymerization of the *closo*-decaborate cluster into biological and pharmacological compounds; such compounds are currently under investigation as anti-inflammatory and antitumor agents.

## Data Availability

Not applicable.

## Supplementary Materials

The following supporting information can be downloaded at: https://www.mdpi.com/article/10.3390/molecules28166076/s1, Figure S1 displays the 11B NMR spectrum for all compounds with carbonyl-closodecaborate, Figures S2, S6, S10 and S20 showcase the 1H NMR spectra for compounds (**2**), (**3**), (**4**) and (**7**) respectively. the ^13^C NMR spectra for all compounds (**2**–**7**) are depicted in Figures S3, S7, S11, S14, S17 and S21. Figures S4, S8, S12, S15, S18 and S22 illustrate the ^31^P NMR spectra for all compounds (**2**–**7**). Figures S5, S9, S13, S16, S19 and S23 provide the mass spectrometry for all compounds (**2**–**7**).
