# Supplementary material for: Selective Functionalization of Carbonyl Closo-Decaborate [2-B10H9CO]− with Building Block Properties via Grignard Reagents
Source: Molecules. 2023 Aug 15;28(16):6076. doi: 10.3390/molecules28166076 (PMC10458521; doi:10.3390/molecules28166076)
Supplement: Supplementary file 1 [file molecules-28-06076-s001.zip › molecules-2518673-supplementary.pdf]

Supporting Information

Selective functionalization of carbonyl closo-decaborate [2-$B_{10}H_9CO]^-$ with building block properties via Grignard reagents

Nadine Mahfouz,[1,2] Fatima Abi-Ghaida,[1,*] Wael Kotob,[1] Ahmad Mehdi,[2,*] and Daoud Naoufal[1,*]

[1] Inorganic and Organometallic Coordination Chemistry Laboratory LCIO, Lebanese University, Faculty of Sciences, Lebanon, dnaoufal@ul.edu.lb, fghaida@ul.edu.lb

[2] Institut Charles Gerhardt ICGM, Université de Montpellier, CNRS, ENSCM, Montpellier, 34090, France, ahmad.mehdi@umontpellier.fr

* Correspondence: fghaida@ul.edu.lb, ahmad.mehdi@umontpellier.fr, dnaoufal@ul.edu.lb

Table of Contents

1. ^{11}B NMR spectrum for all compounds with carbonyl-closodecaborate(1) 2
2. Product $(PPh_4)(MgBr)[2\text{-}B_{10}H_9C(O)CH_2CH_3]$ (2) 3
   2.1. ^{1}H NMR Spectrum 3
   2.2. ^{13}C NMR spectrum 4
   2.3. ^{31}P NMR spectrum 5
   2.4. Mass spectrometry ESI/MS 5
3. Product (PPh_4) $(MgBr)[2\text{-}B_{10}H_9C(O)(CH_2)_4CH_3]$ (3) 6
   3.1. ^{1}H NMR Spectrum 6
   3.2. ^{13}C NMR Spectrum 7
   3.3. ^{31}P NMR spectrum 8
   3.4. Mass spectrometry ESI/MS 8
4. Product (PPh_4) $(MgCl)[2\text{-}B_{10}H_9C(O)C_3H_7]$ (4) 9
   4.1. ^{1}H NMR spectrum 9
   4.2. ^{13}C NMR spectrum 10
   4.3. ^{31}P NMR spectrum 11
   4.4. Mass spectrometry ESI/MS 11
5. Product (PPh_4) $(MgCl)[2\text{-}B_{10}H_9C(O)CH_2CH{=}CH_2]$ (5) 12
   5.1. ^{13}C NMR spectrum 12
   5.2. ^{31}P NMR spectrum 13

5.3. Mass spectrometry ESI/MS 13
6. Product (PPh_4) (MgBr)[2-$B_{10}H_9C(O)CH{=}CH_2$] (6) 14
6.1. ^{13}C NMR spectrum 14
6.2. ^{31}P NMR spectrum 15
6.3. Mass spectrometry ESI/MS 15
7. Product (PPh_4) (MgBr)[2-$B_{10}H_9C(O)C{\equiv}CCH_3$] (7) 16
7.1. ^{1}H NMR spectrum 16
7.2. ^{13}C NMR spectrum 17
7.3. ^{31}P NMR spectrum 18
7.4. Mass spectrometry ESI/MS 18

1. ^{11}B NMR spectrum for all compounds with carbonyl-closodecaborate(1)

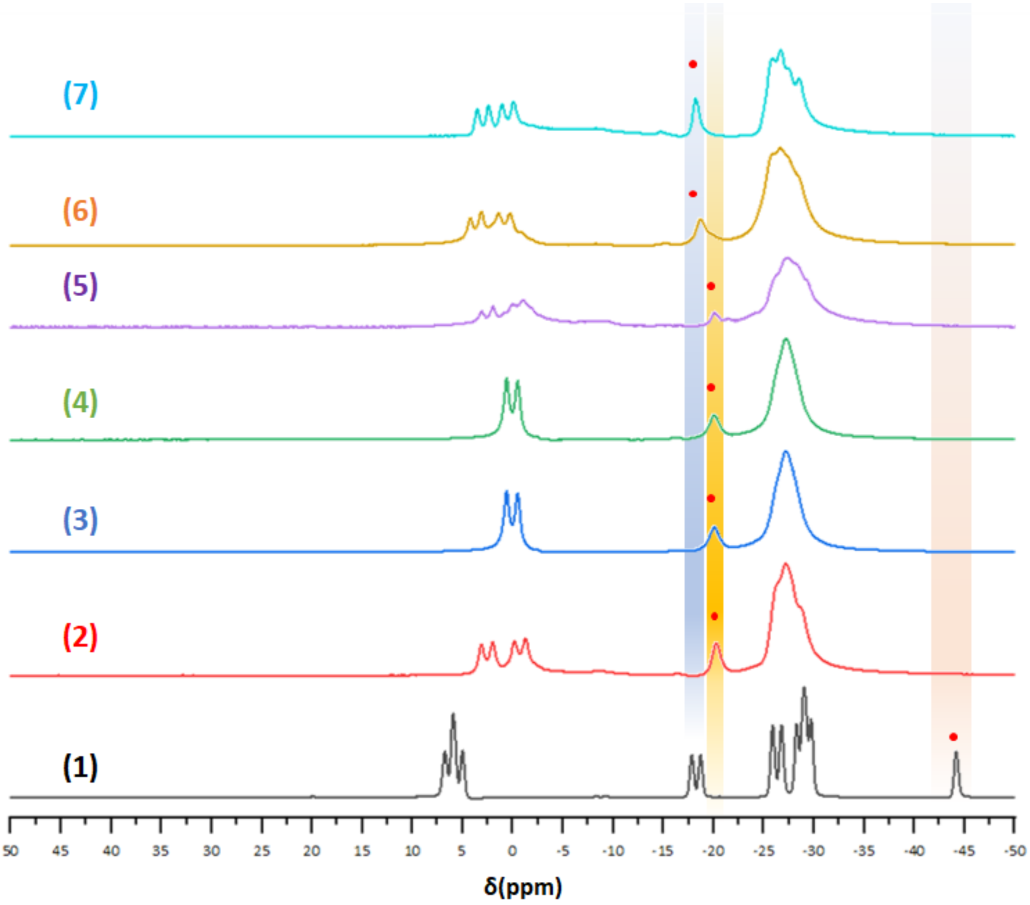

Figure S1. ^{11}B NMR spectrum for all compounds with carbonyl-closodecaborate (1).

2. Product $(PPh_4)(MgBr)[2\text{-}B_{10}H_9C(O)CH_2CH_3]$ (2)

2.1. ^{1}H NMR Spectrum

^{1}H NMR (δ ppm, DMSO-d$_6$): 0.65 (3 H, d, CH_3), 2.23 (2 H, quadruplet, CH_2), 7.5-7.8 (20 H, m, H of PPh_4^+).

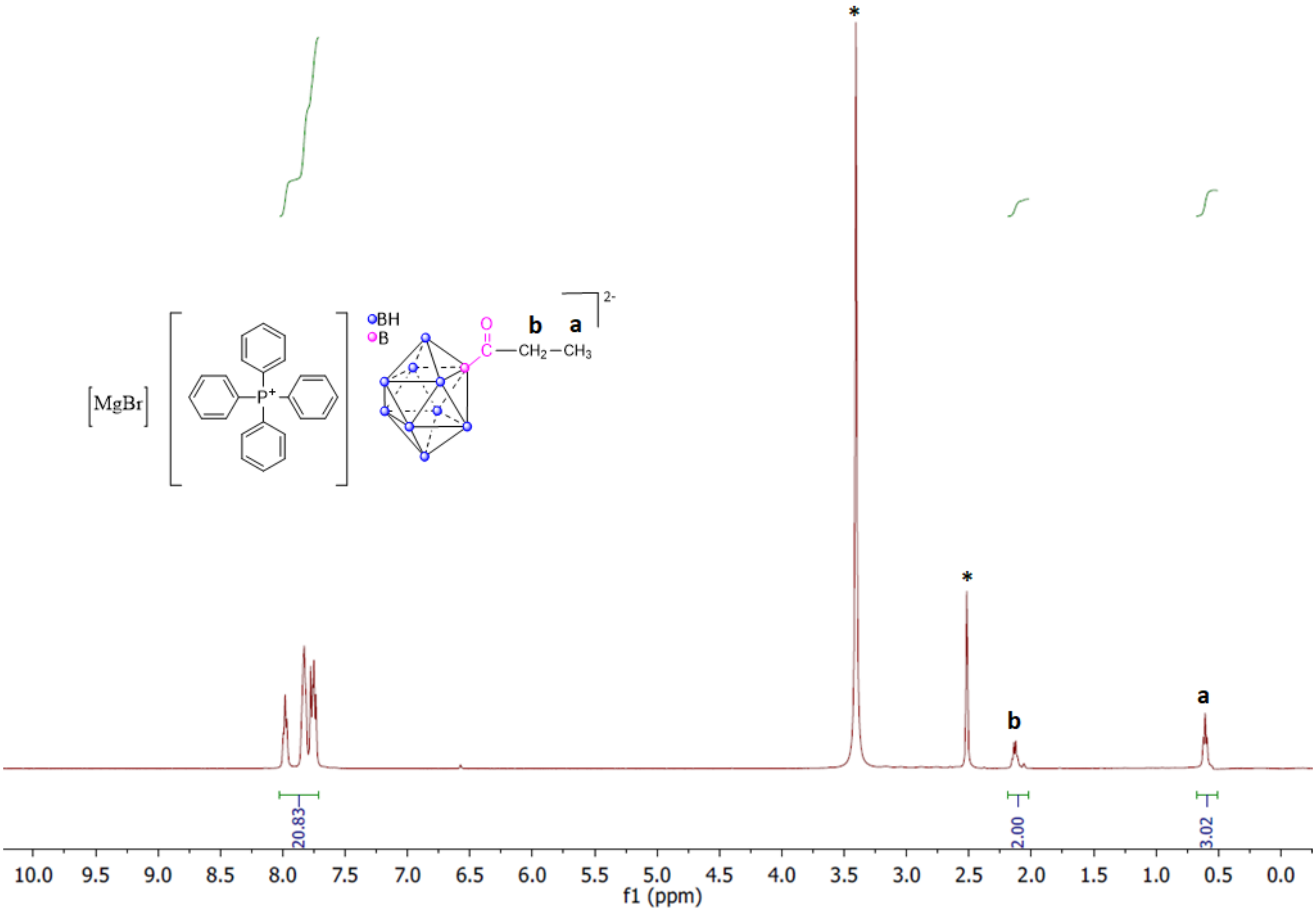

Figure S2. ^{1}H NMR spectrum of Product (2) in DMSO-d$_6$.

2.2. ^{13}C NMR spectrum

^{13}C NMR (δ ppm, DMSO-d$_6$): 157.30 (a), 134.16 (g), 133.32 (f), 129.31 (e), 116.49(d), 7.25 (c), 1.13(b)

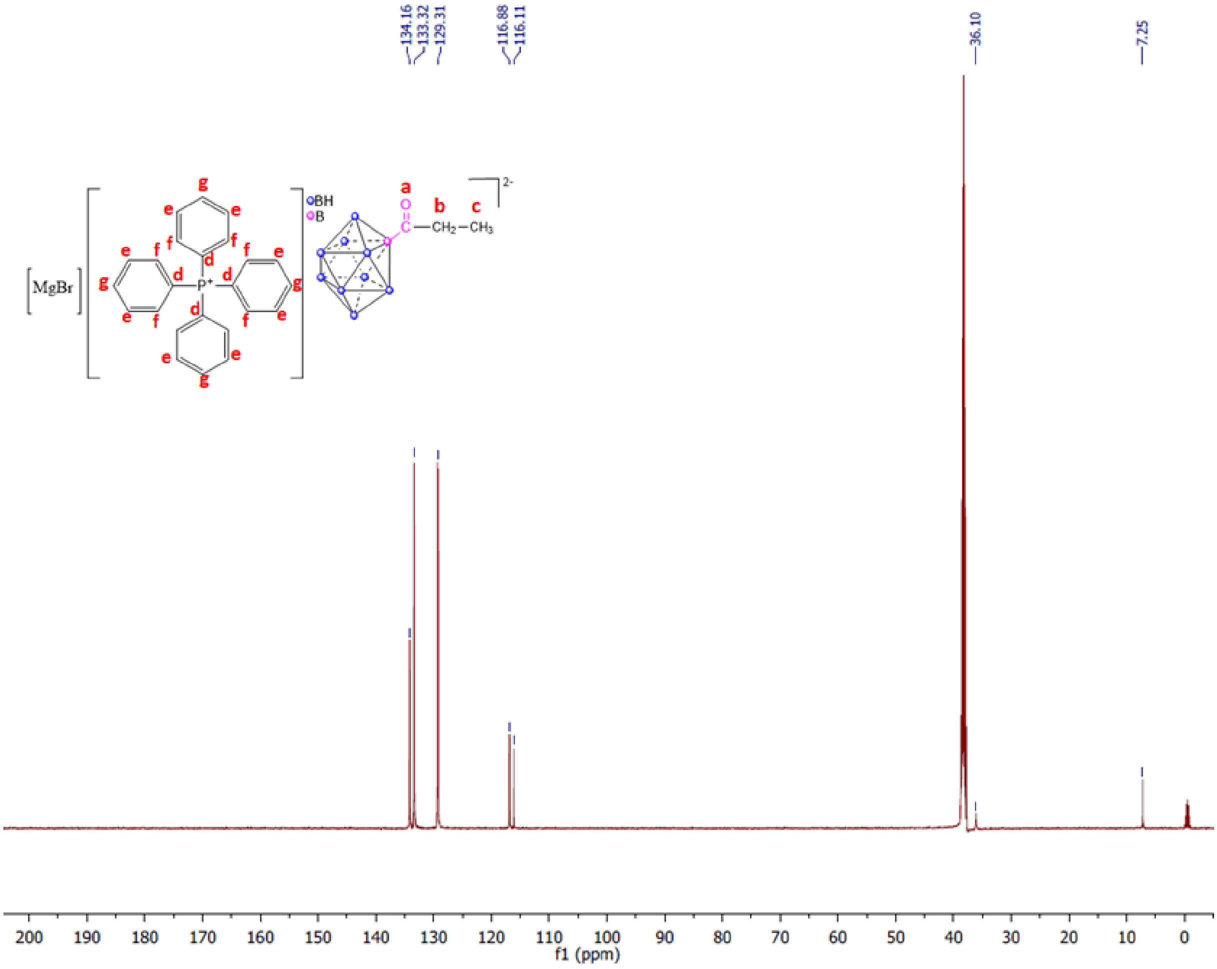

Figure S3. **^{13}C NMR Spectrum of product (2). Attributed carbons (a to g) are annotated on the structure.**

2.3. ^{31}P NMR spectrum

^{31}P NMR (DMSO-d_6): 22.59

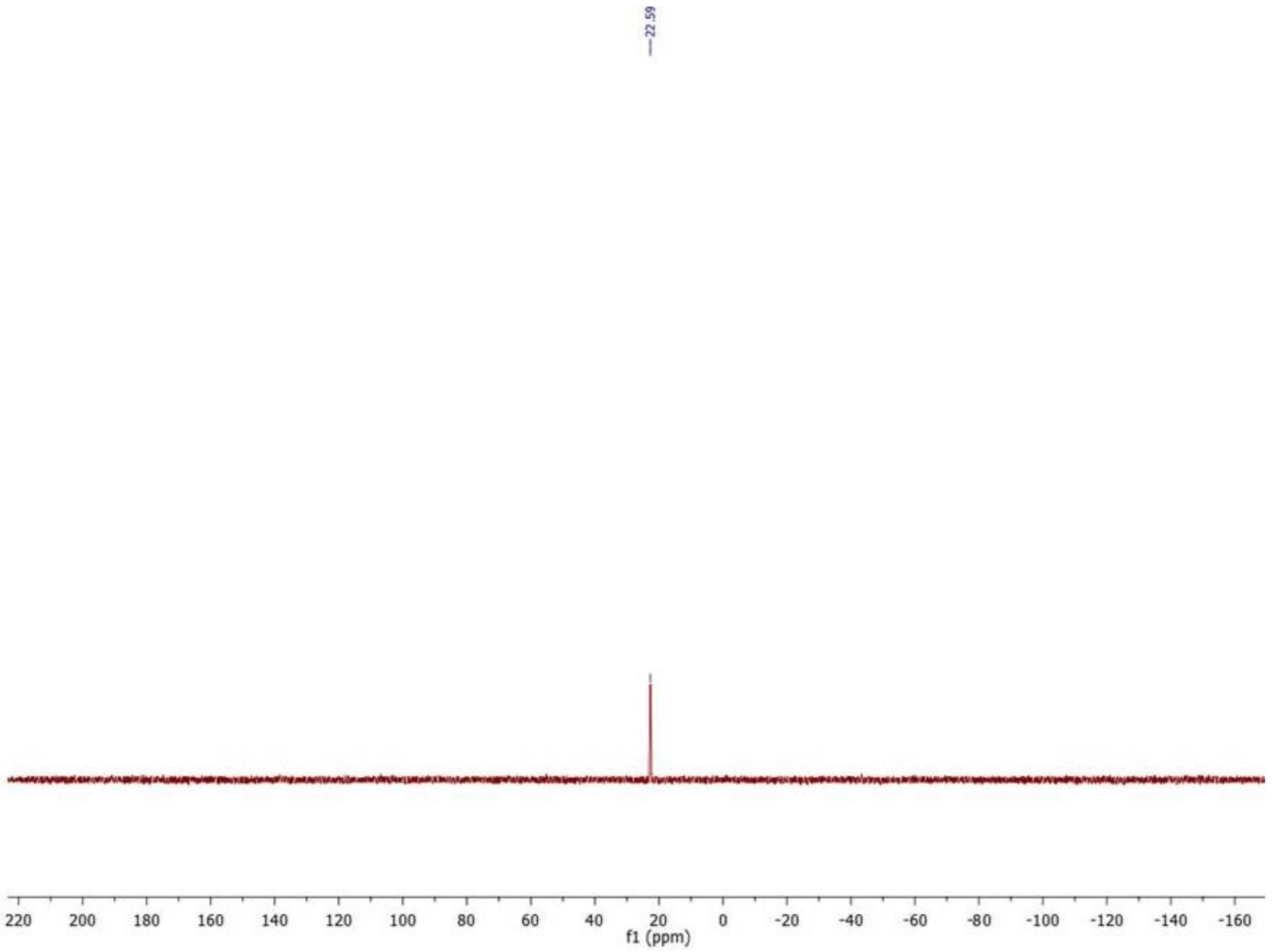

Figure S4. ^{31}P NMR spectrum of product (2).

2.4. Mass spectrometry ESI/MS

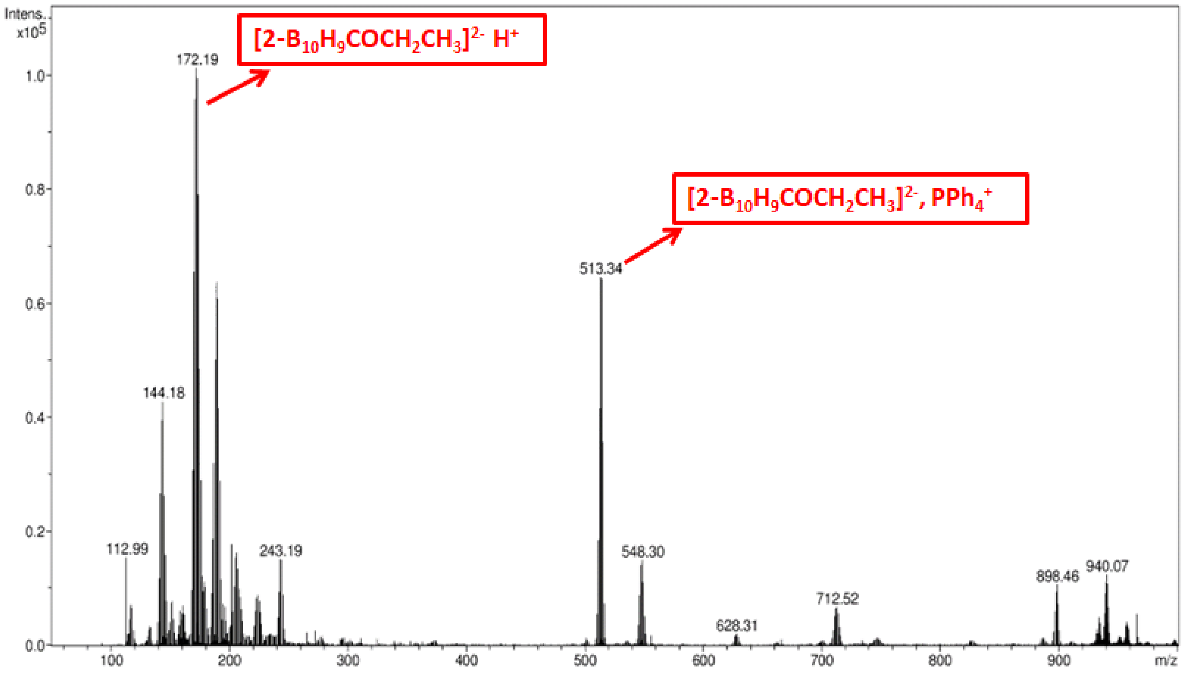

Figure S5. ESI/MS spectra of the product (2).

3. Product $(PPh_4)(MgBr)[2\text{-}B_{10}H_9C(O)(CH_2)_4CH_3]$ (3)

3.1. ^1H NMR Spectrum

^{1}H NMR (δ ppm, DMSO-d_6): 0.75 (3H, t, CH_3), 1.10 (2H, sextuplet, CH_2), 1.25 (4H, quintuplet, $2CH_2$), 2.15 (2H, t, CH_2), 7.5-7.8 (20H, m, H of PPh_4^+)

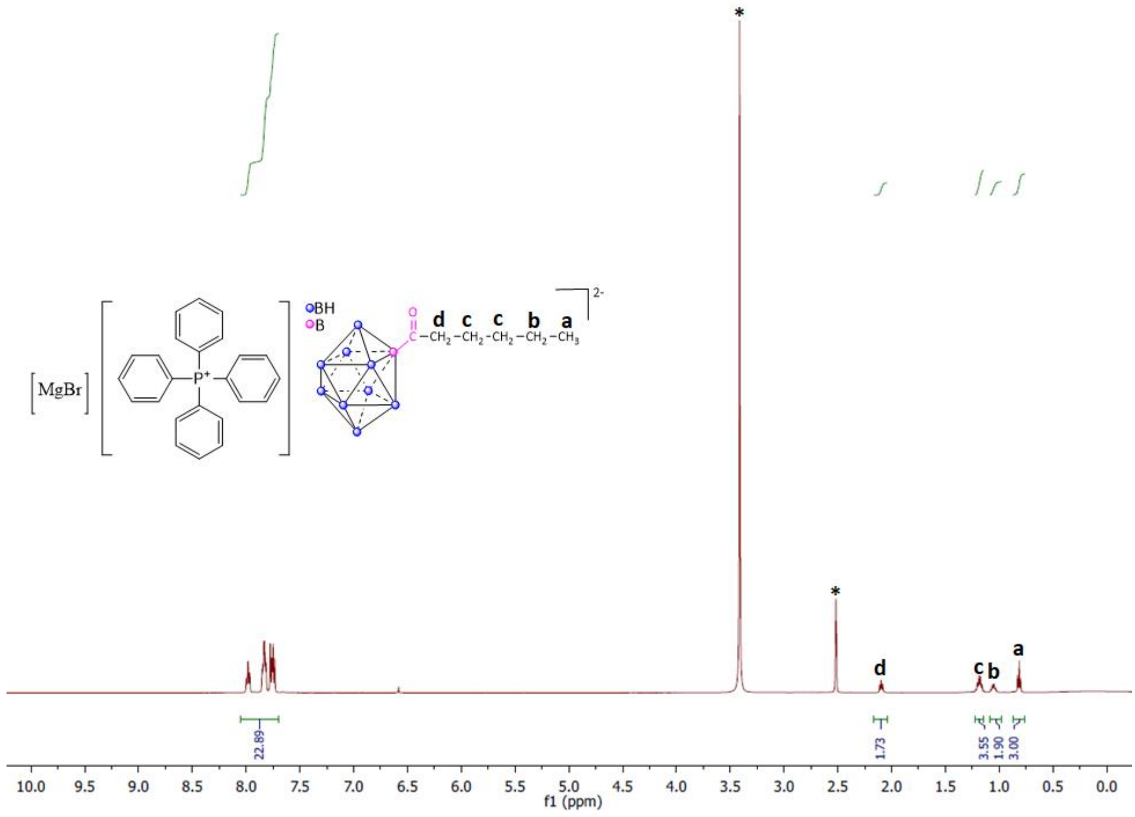

Figure S6. ^{1}H NMR spectrum of Product 3 in DMSO-d6.

3.2. ^{13}C NMR Spectrum

^{13}C NMR (δ ppm, DMSO-d$_6$): 166.35 (a), 135.85 (g), 135.01 (f), 131.01 (e), 118.14 (d),32.08 (b), 24.01 (c), 22.73 (h), 14.50 (i).

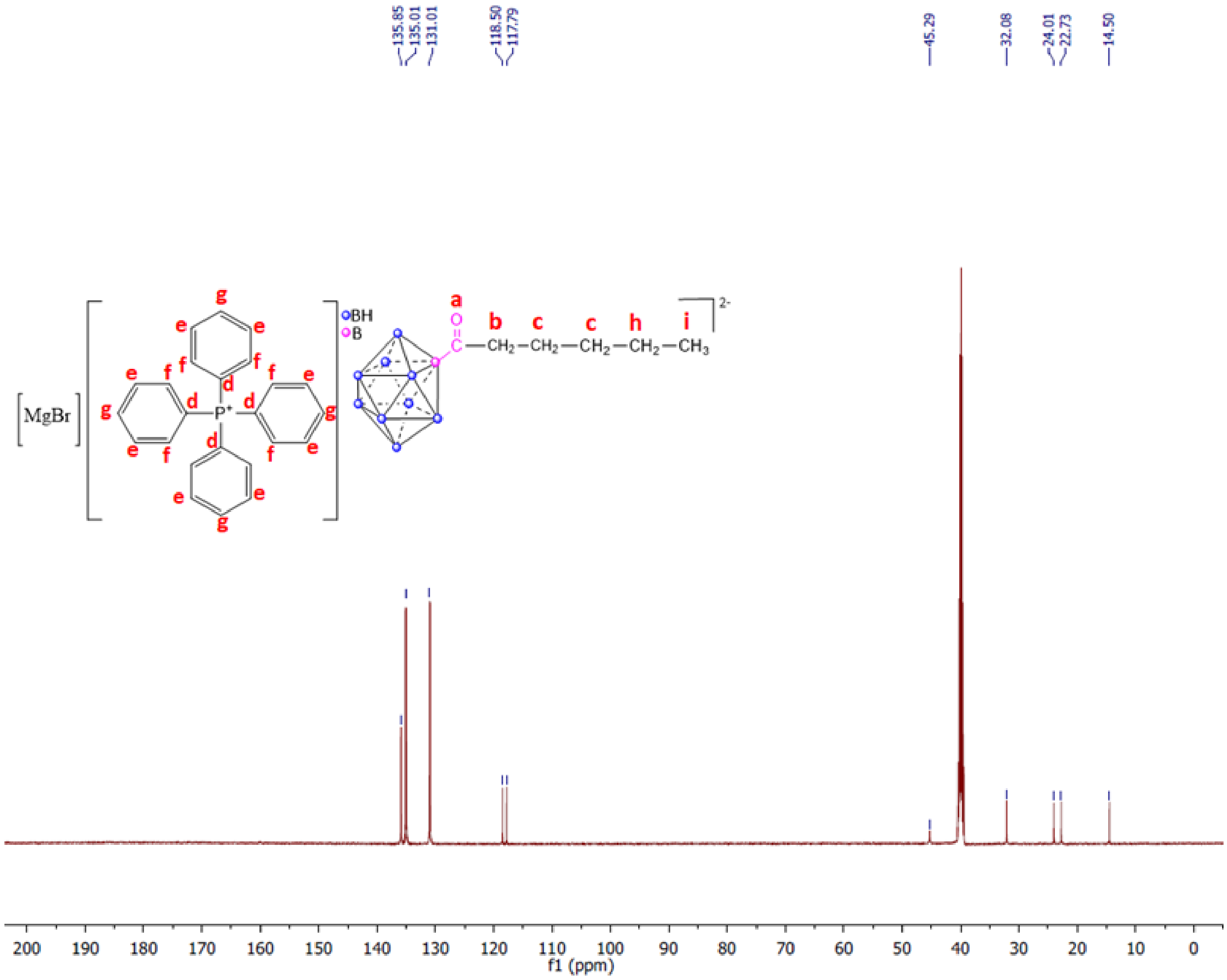

Figure S7. ^{13}C NMR Spectrum of product (3). Attributed carbons (a to i) are annotated on the structure.

3.3. ^{31}P NMR spectrum

^{31}P NMR (DMSO-d_6): 22.13

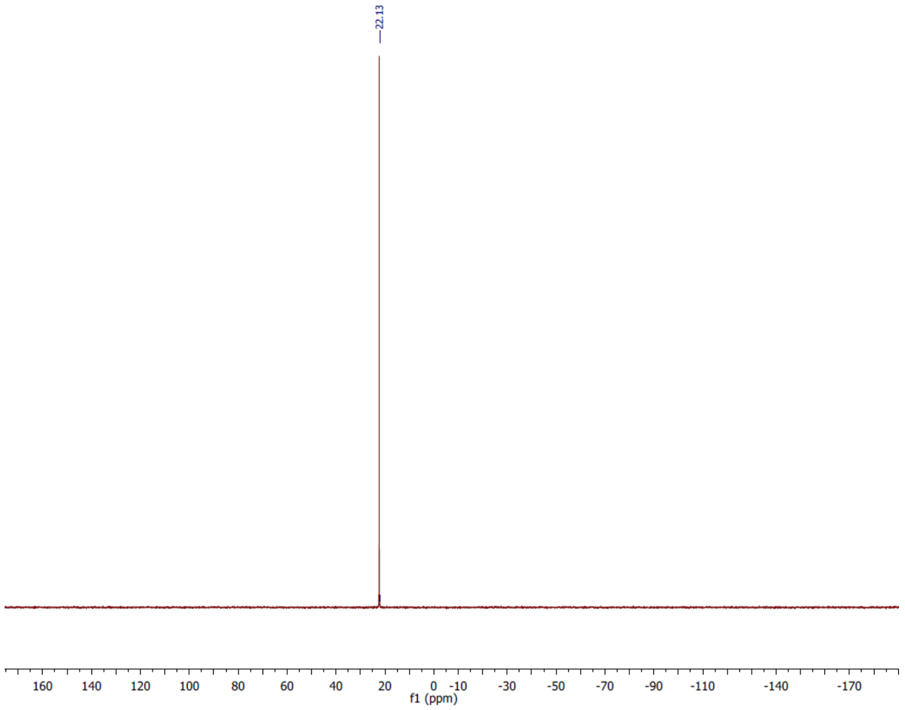

Figure S8. ^{31}P spectrum of the product (3).

3.4. Mass spectrometry ESI/MS

Mass spectrometry m/z (3) = 216

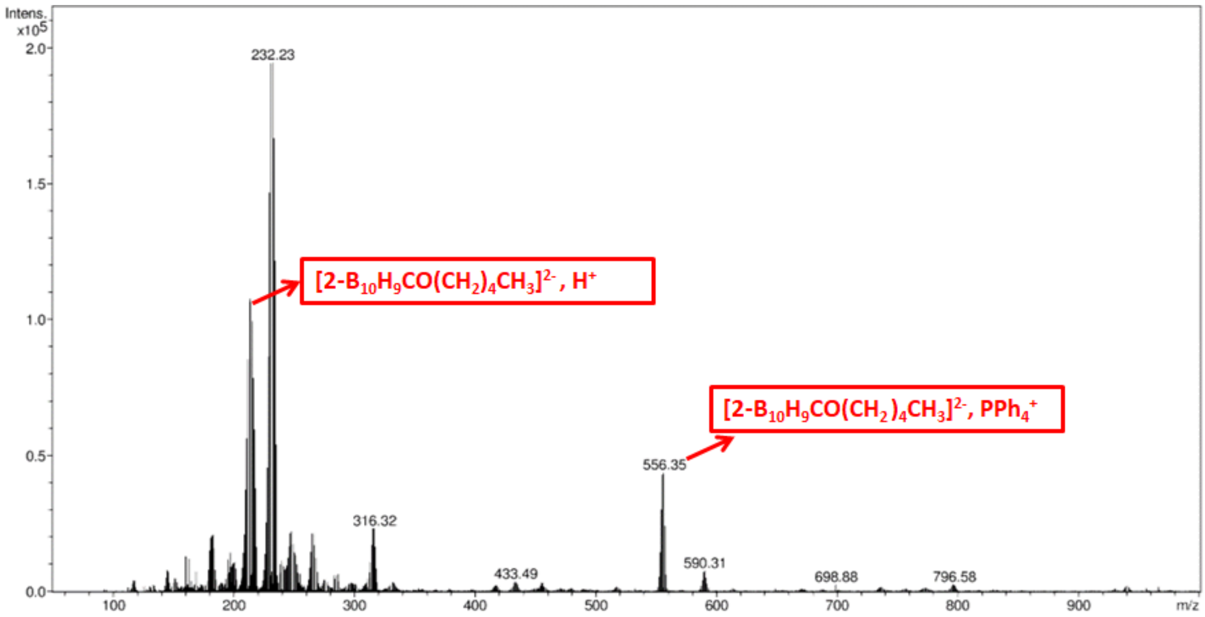

Figure S9. Mass spectrometry ESI/MS of the product (3).

4. Product (PPh_4) ($MgCl$)[2-$B_{10}H_9C(O)C_3H_7$] (4)

4.1. ^{1}H NMR spectrum

^{1}H NMR (δ ppm, DMSO-d_6): 0.68 (6H, d, $2CH_3$), 2.65 (1H, septuplet, CH), 7.51-8.04 (20H, m, H of PPh_4^+)

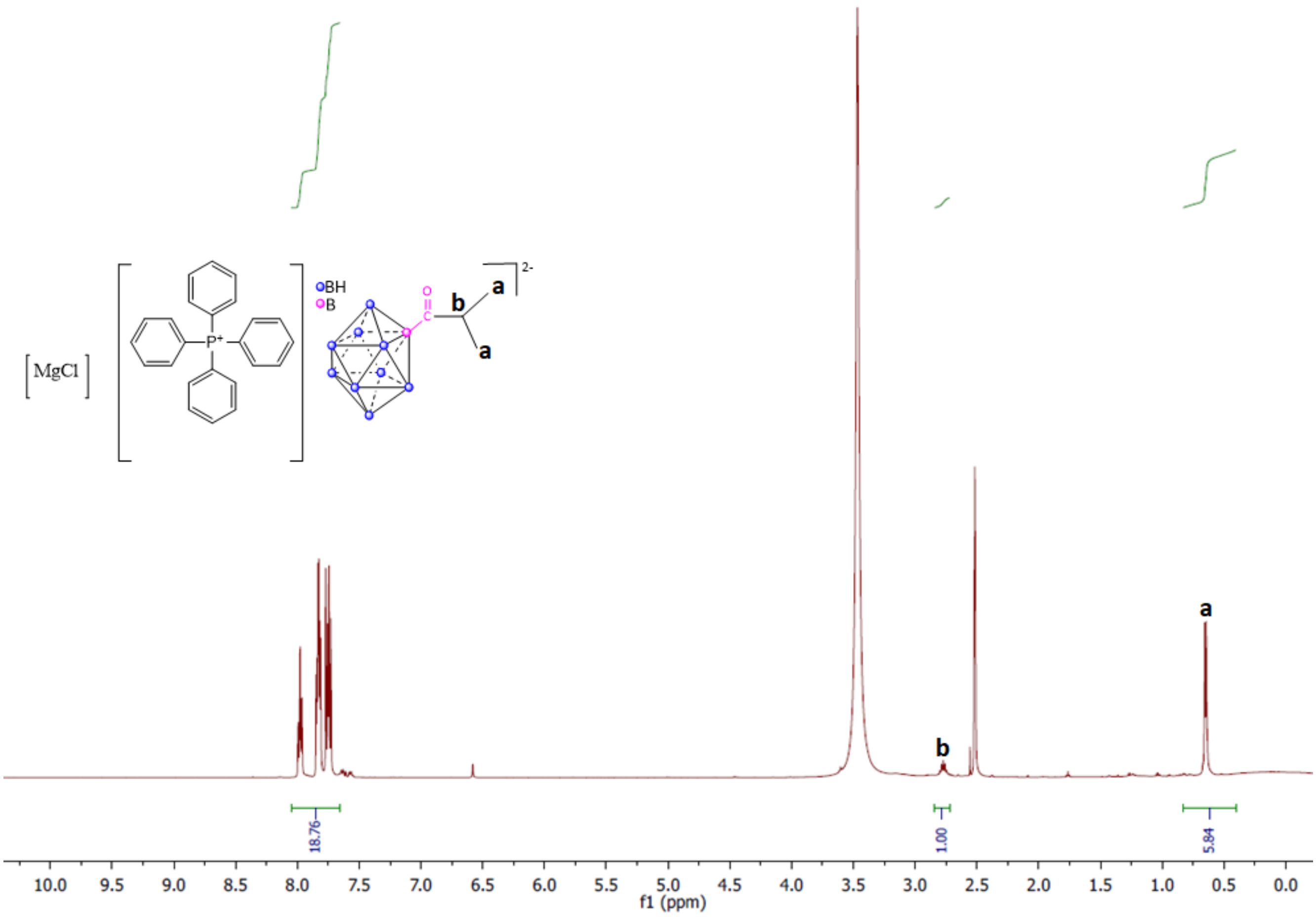

Figure S10. **^{1}H NMR spectrum of Product (4) in DMSO-d_6.**

4.2. ^{13}C NMR spectrum

^{13}C NMR (δ ppm, DMSO-d_6): 135.86 (g), 135.00 (f), 131.01 (e),118.13 (d), 19.79 (c).

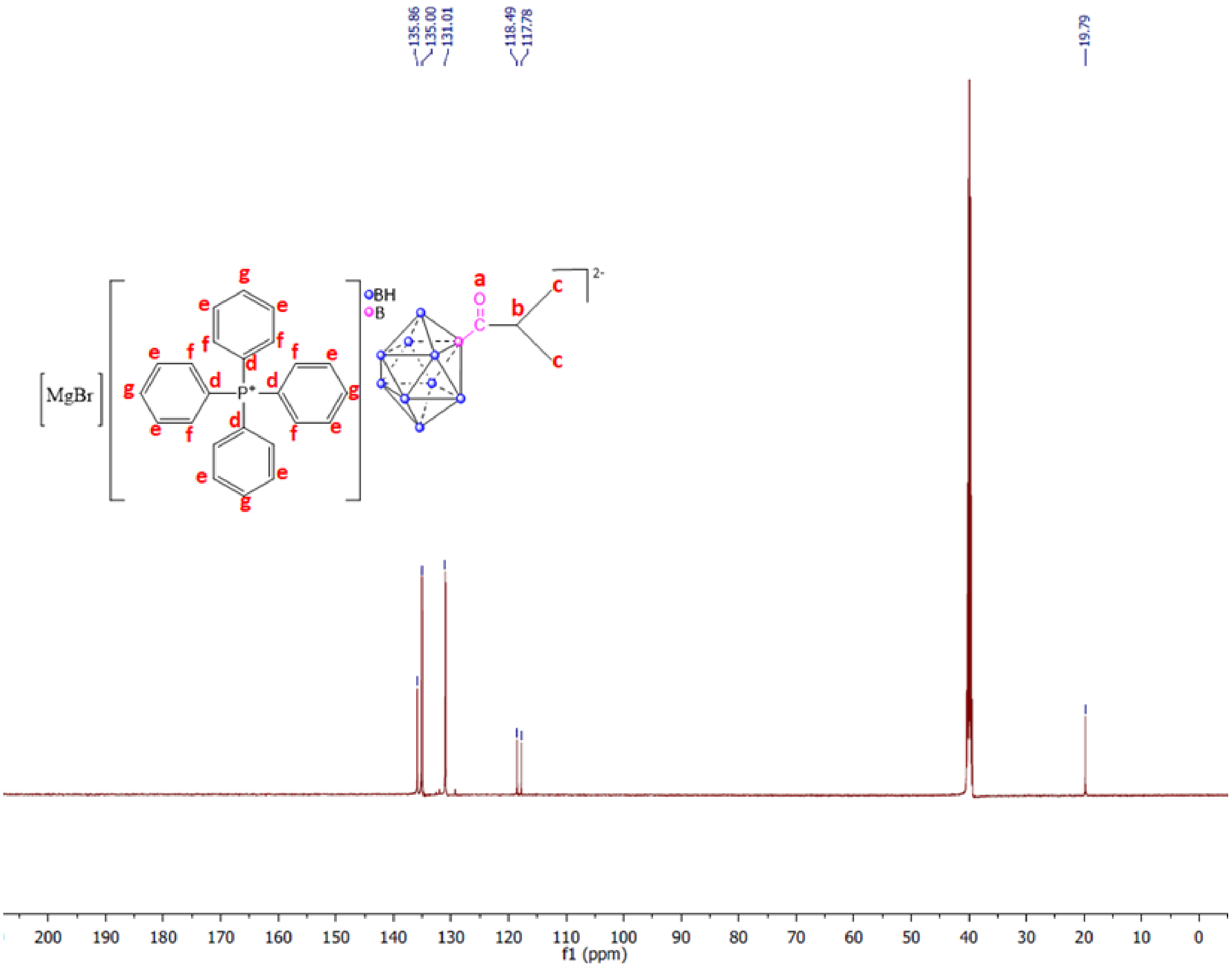

Figure S11. ^{13}C NMR Spectrum of product (4). Attributed carbons (a to g) are annotated on the structure.

4.3. ^{31}P NMR spectrum

^{31}P NMR (DMSO-d_6): 22.59

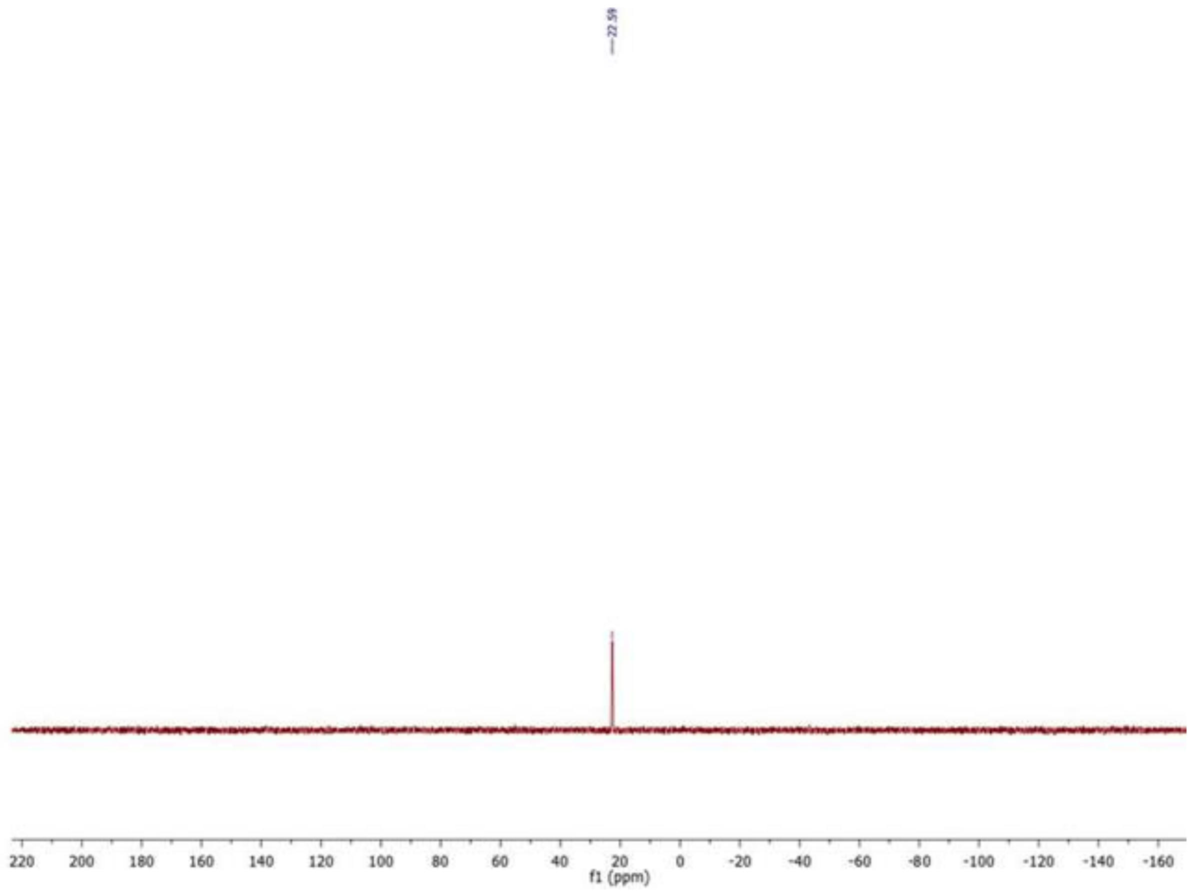

Figure S12. ^{31}P NMR spectrum of the product (4).

4.4. Mass spectrometry ESI/MS

Mass spectrometry (ESI): m/z (4) = 188

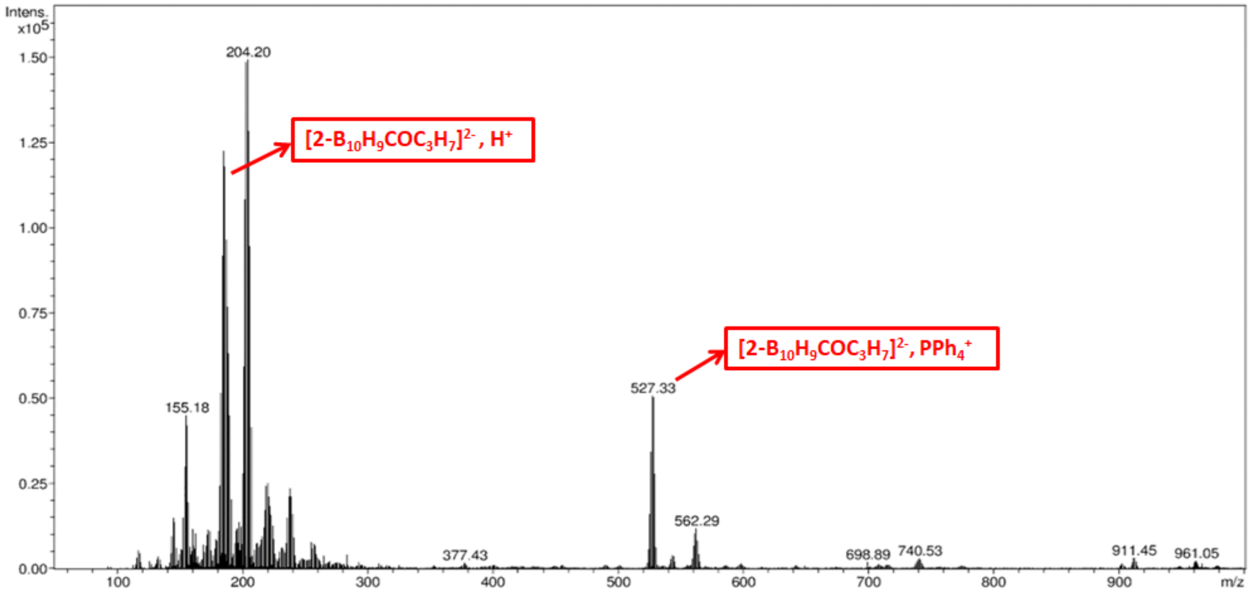

Figure S13. Mass spectrometry ESI/MS of the product (4).

5. Product ($(PPh_4)(MgCl)[2\text{-}B_{10}H_9C(O)CH_2CH{=}CH_2]$) (5)

5.1. ^{13}C NMR spectrum

^{13}C NMR (δ ppm, DMSO-d_6): 135.86(g), 135.08 (h), 131.01(f),130.84 (e), 118.13 (d), 60.55 (b)

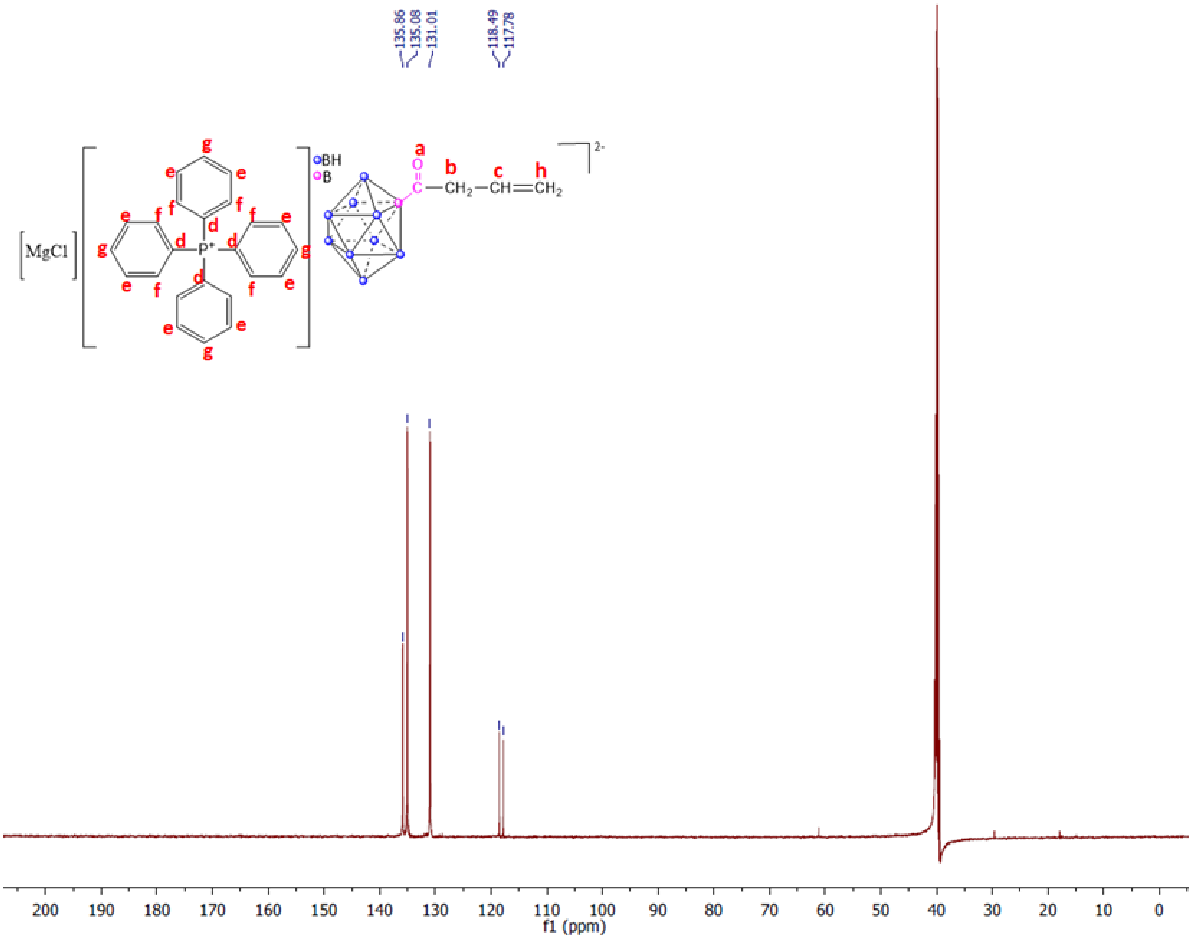

Figure S14. ^{13}C NMR Spectrum of product (5). Attributed carbons (a to h) are annotated on the structure.

5.2. ^{31}P NMR spectrum

^{31}P NMR (DMSO-d_6): 21.64

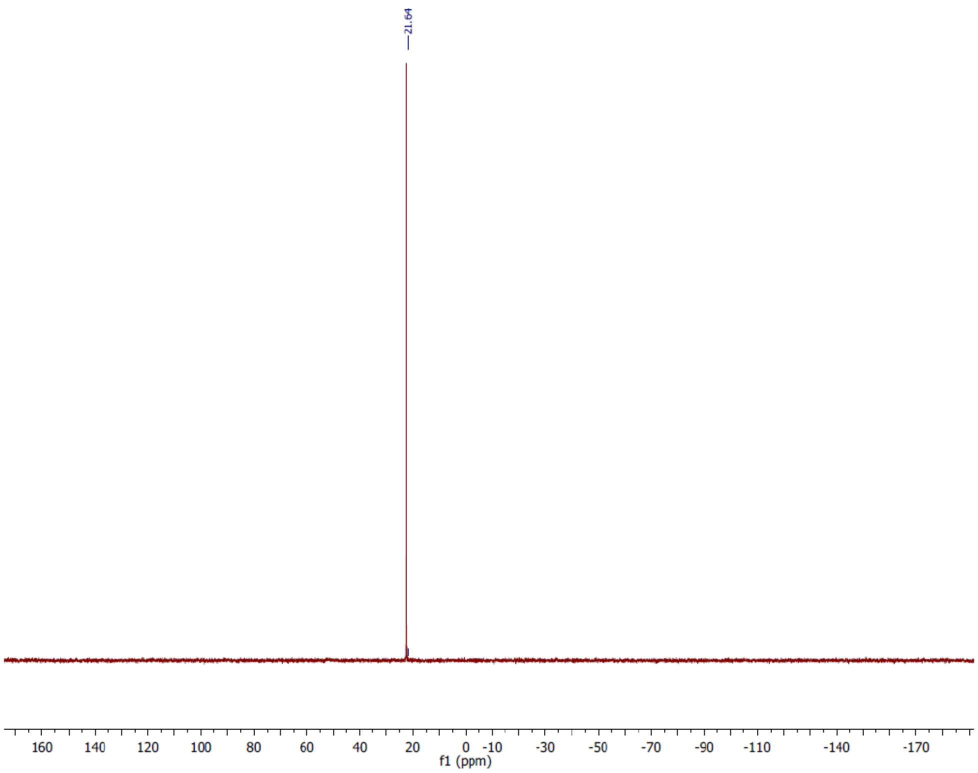

Figure S15. ^{31}P NMR spectrum of product (5).

5.3. Mass spectrometry ESI/MS

Mass spectrometry (ESI): m/z (5)= 185

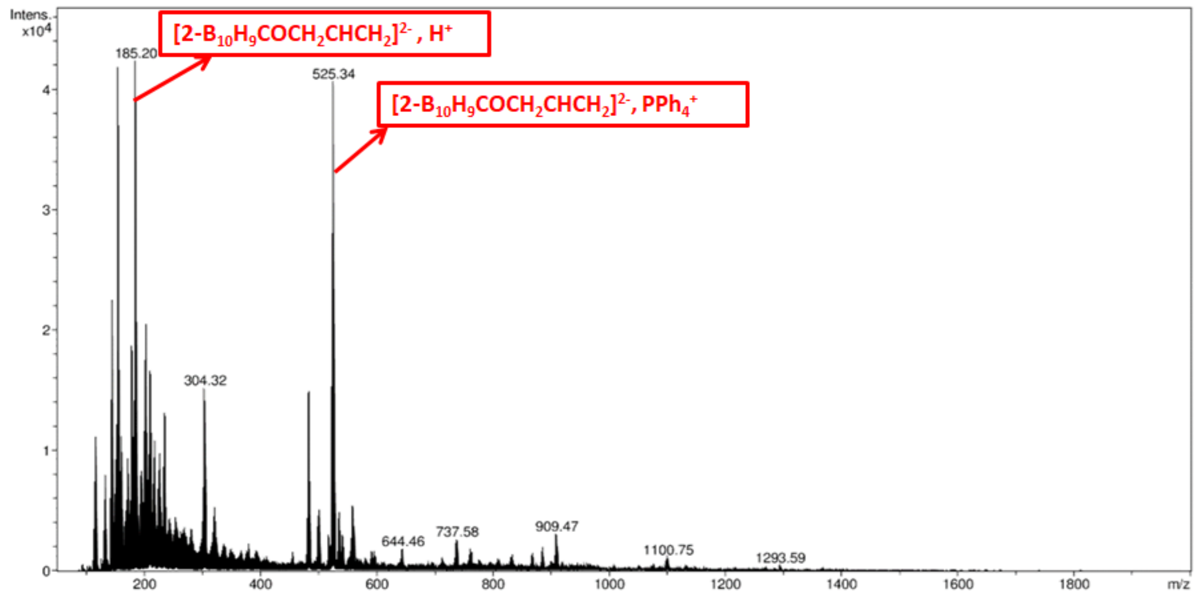

Figure S16. Mass spectrometry ESI/MS of the product (5).

6. Product (PPh$_4$) (MgBr)[2-B$_{10}$H$_9$C(O)CH=CH$_2$] (6)

6.1. ^{13}C NMR spectrum

^{13}C NMR (δ ppm, DMSO-d$_6$): 175.56 (a), 135.85(b), 135.09(g),131.01(f),118.14 (d)

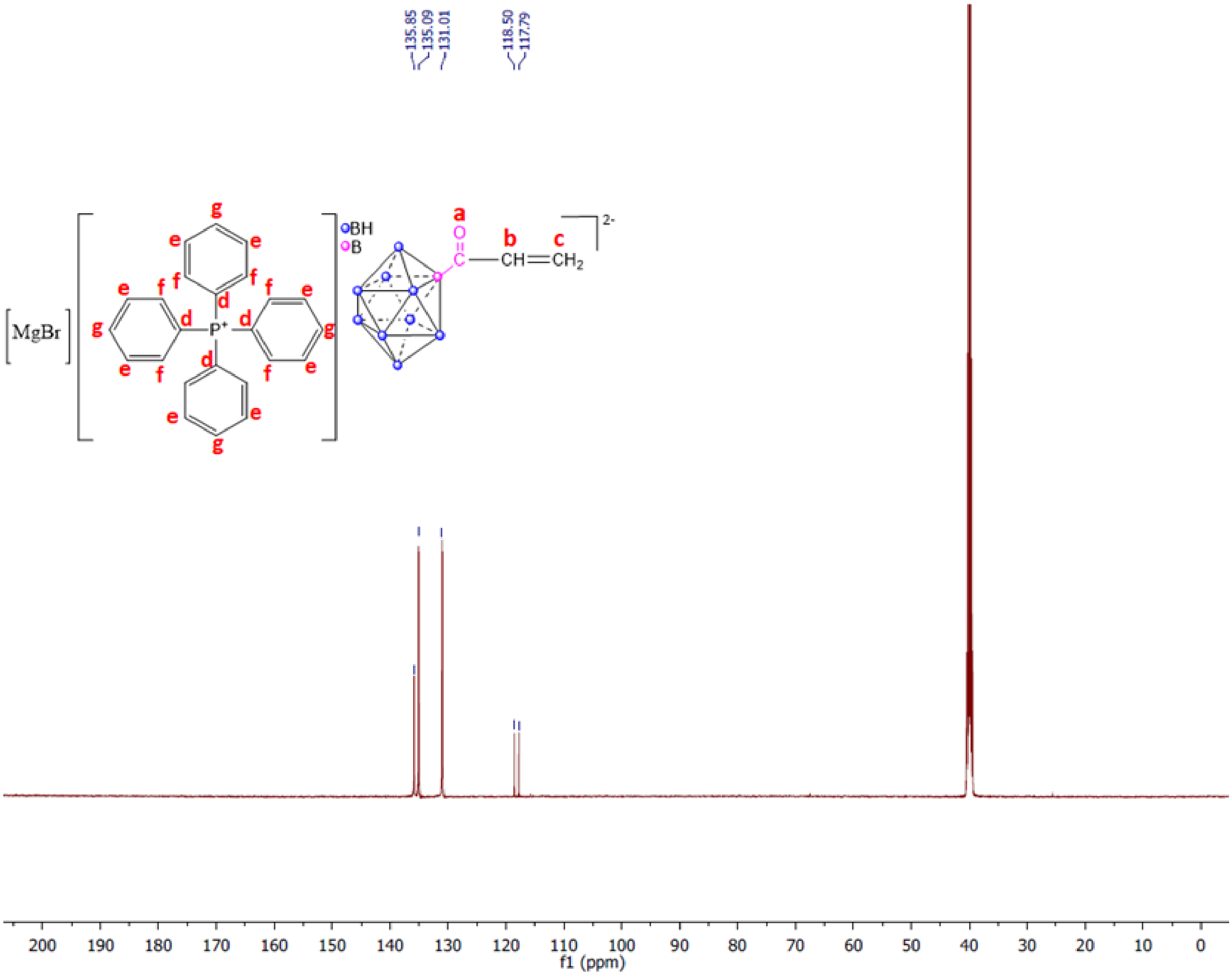

Figure S17. ^{13}C NMR Spectrum of product (6). Attributed carbons (a to g) are annotated on the structure.

6.2. ^{31}P NMR spectrum

^{31}P NMR (DMSO-d_6): 22.14.

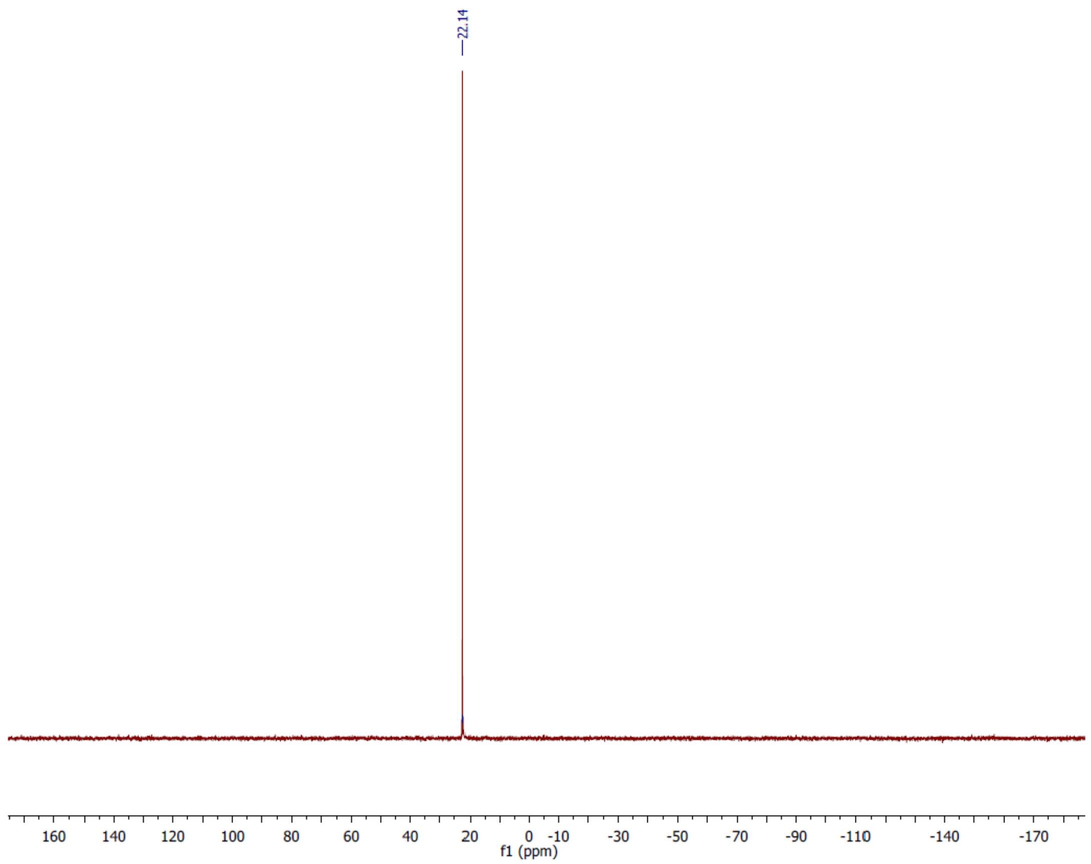

Figure S18. ^{31}P NMR Spectrum of the product (6).

6.3. Mass spectrometry ESI/MS

Mass spectrometry (ESI): m/z (6) = 172.

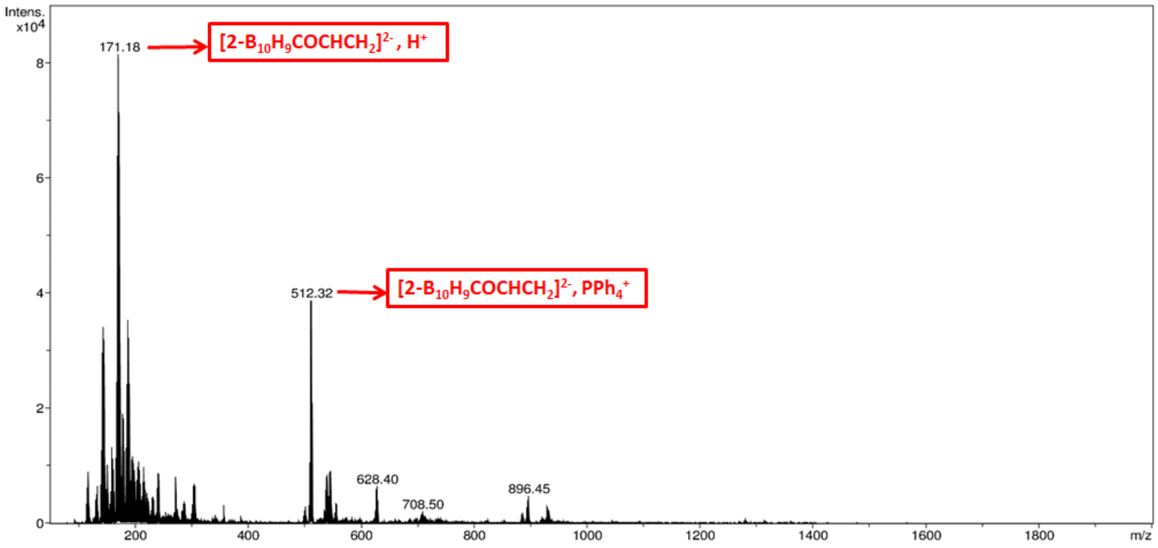

Figure S19. Mass spectrometry ESI/MS of the product (6).

7. Product $(PPh_4)(MgBr)[2\text{-}B_{10}H_9C(O)C{\equiv}CCH_3]$ (7)

7.1. ^{1}H NMR spectrum

^{1}H NMR (δ ppm, DMSO-d_6): 1.75 (3 H, s, CH_3), 7.5-7.8 (20 H, m H of PPh_4^+).

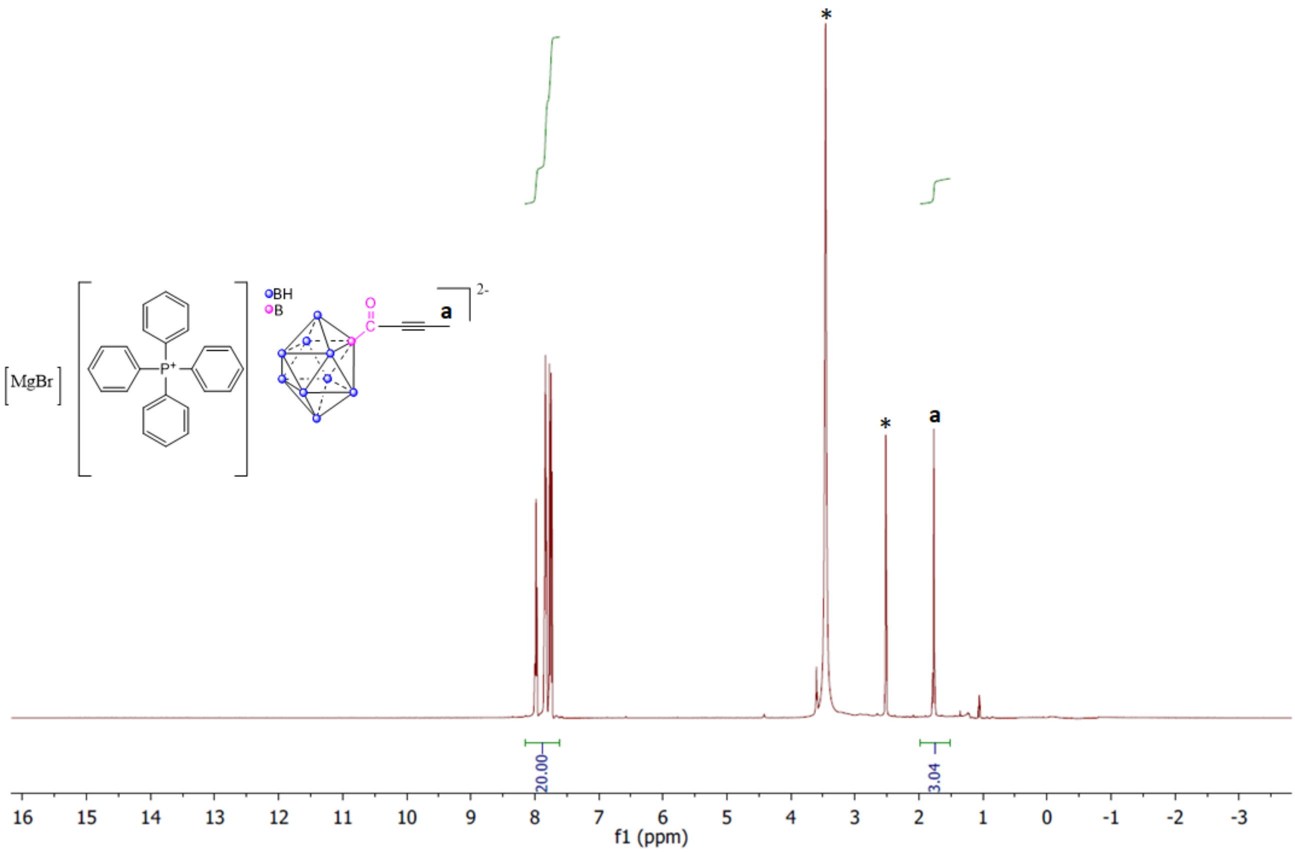

Figure S20. ^{1}**H NMR spectrum of Product (7) in DMSO-d_6.**

7.2. ^{13}C NMR spectrum

^{13}C NMR (δ ppm, DMSO-d_6): 171.20, 135.87(g), 135.00(f), 131.02(e), 118.13(d), 82.95(c), 67.50(b), 25.66(a)

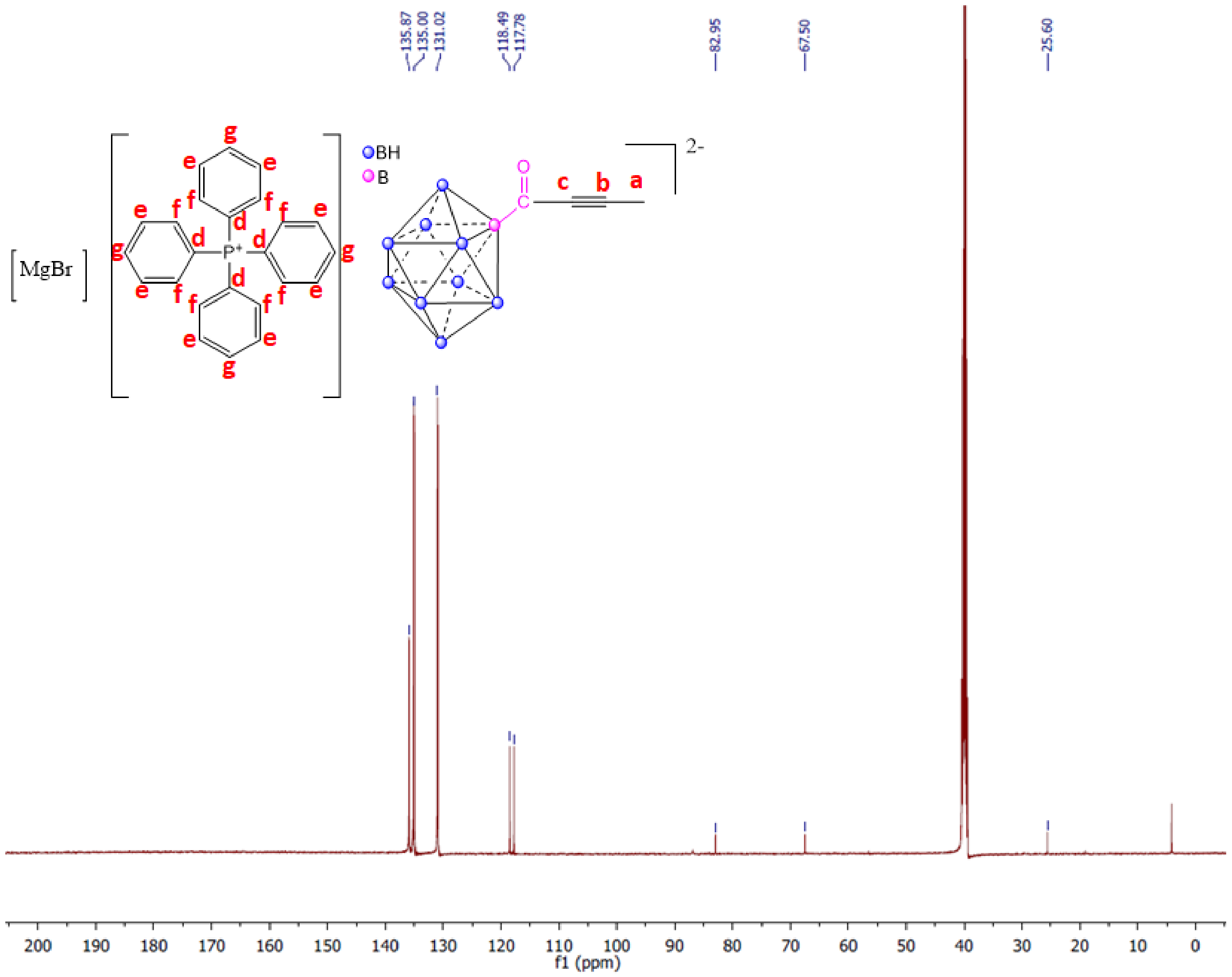

Figure S21. **^{13}C NMR spectrum of the compound (7). Attributed carbons (a to g) are annotated on the structure.**

7.3. ^{31}P NMR spectrum

^{31}P NMR (DMSO-d_6): 22.13.

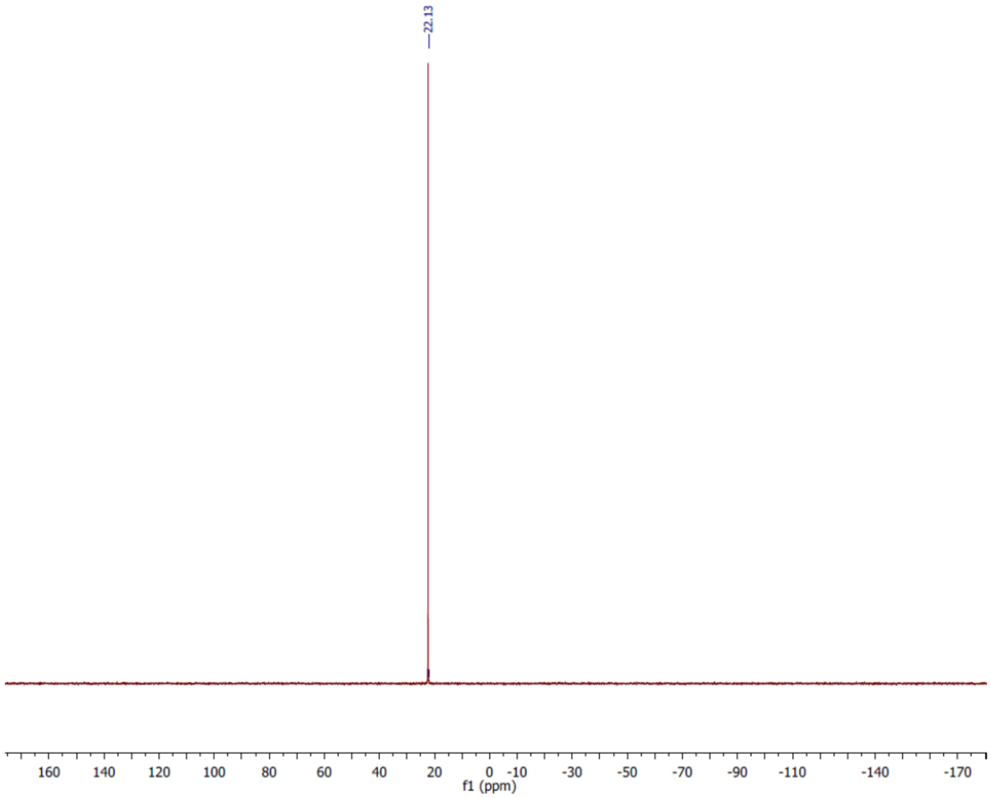

Figure S22. ^{31}P NMR spectrum of the product (7*).*

7.4. Mass spectrometry ESI/MS

Mass spectrometry (ESI): m/z (6) = 184

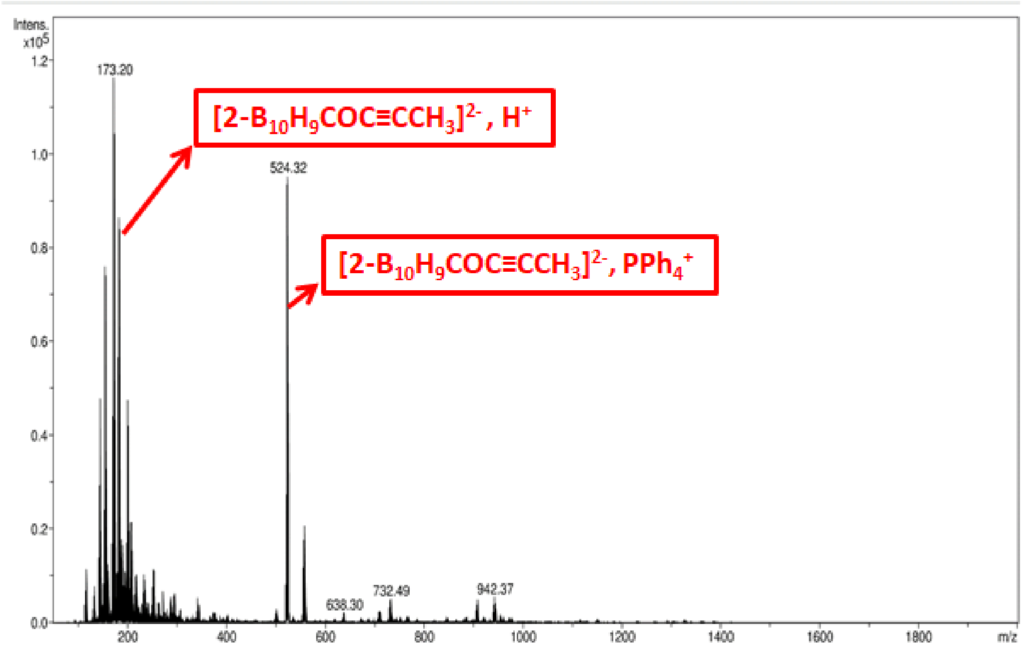

Figure S23. Mass spectrometry ESI/MS of the product (7).
